# Electromagnetic power of lightning superbolts from Earth to space

**DOI:** 10.1038/s41467-021-23740-6

**Published:** 2021-06-11

**Authors:** J.-F. Ripoll, T. Farges, D. M. Malaspina, G. S. Cunningham, E. H. Lay, G. B. Hospodarsky, C. A. Kletzing, J. R. Wygant, S. Pédeboy

**Affiliations:** 1 CEA, DAM, DIF, Arpajon, France; 2 UPS, CEA, LMCE, Bruyères-le-Châtel, France; 3 Department of Astrophysical and Planetary Sciences, University of Colorado, Boulder, CO USA; 4 Laboratory for Atmospheric and Space Physics, University of Colorado, Boulder, CO USA; 5 Space Science and Applications Group, Los Alamos National Laboratory, Los Alamos, NM USA; 6 Space and Remote Sensing Group, Los Alamos National Laboratory, Los Alamos, NM USA; 7 Department of Physics and Astronomy, University of Iowa, Iowa City, IA USA; 8 School of Physics and Astronomy, University of Minnesota, Minneapolis, MN USA; 9 Météorage, Pau, France

**Keywords:** Atmospheric dynamics, Magnetospheric physics

## Abstract

Lightning superbolts are the most powerful and rare lightning events with intense optical emission, first identified from space. Superbolt events occurred in 2010-2018 could be localized by extracting the high energy tail of the lightning stroke signals measured by the very low frequency ground stations of the World-Wide Lightning Location Network. Here, we report electromagnetic observations of superbolts from space using Van Allen Probes satellite measurements, and ground measurements, and with two events measured both from ground and space. From burst-triggered measurements, we compute electric and magnetic power spectral density for very low frequency waves driven by superbolts, both on Earth and transmitted into space, demonstrating that superbolts transmit 10-1000 times more powerful very low frequency waves into space than typical strokes and revealing that their extreme nature is observed in space. We find several properties of superbolts that notably differ from most lightning flashes; a more symmetric first ground-wave peak due to a longer rise time, larger peak current, weaker decay of electromagnetic power density in space with distance, and a power mostly confined in the very low frequency range. Their signal is absent in space during day times and is received with a long-time delay on the Van Allen Probes. These results have implications for our understanding of lightning and superbolts, for ionosphere-magnetosphere wave transmission, wave propagation in space, and remote sensing of extreme events.

## Abstract (web summary)

Superbolts are powerful, rare lightning events. Here, the authors show simultaneous satellite and ground measurements of a superbolt, and demonstrate different properties of superbolts and lightnings.

## Introduction

**Fig. 1:**
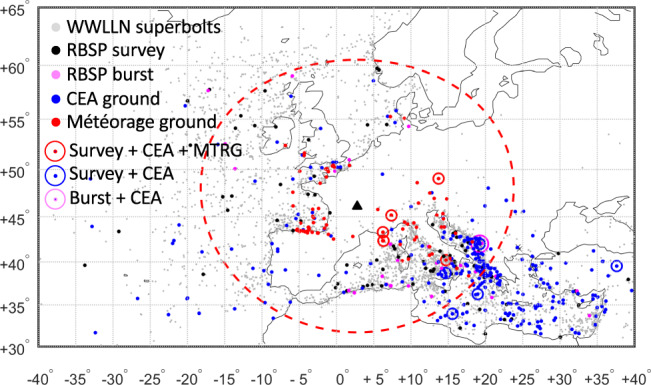
Map of all European WWLLN-detected superbolts in 2012–2018. All European (gray circles) WWLLN-detected superbolts in 2012–2018, among which we highlight the superbolts measured by Van Allen Probes (RBSP) in (black circles) survey and (pink circles) burst modes. WWLLN superbolts seen in coincidence by (blue circles) ECLAIR and (red circles) both ECLAIR and Météorage ground measurements. (large pink circles) Synchronized observations from both space and ground with WWLLN, Van Allen Probes burst and ECLAIR. The red dashed circle defines a 1500 km radius centered on the black triangle indicating the location of one of the ECLAIR stations used here. The *x* and *y* axes are longitude and latitude, respectively. The map itself is made with ©Matlab Mapping Toolbox.

Lightning superbolts are rare and extreme events that were first identified from optical stroke data measured by a photometer on board the Vela satellites^1,2^, yielding between 10^11^ and 10^13^ W per stroke. With such high radiated power, the temperature in the core channel of the stroke must exceed the commonly accepted maximum temperature of the lightning return stroke^3,4^ (i.e., ~3 × 10^5^ K)^5^, creating a debate concerning our current understanding of the energy balance in a lightning discharge^3,6^. Recently, radio frequency (RF) superbolts were geographically localized^7^ using the tail of the occurrence distribution in lightning energy^8^, defined as above 1 MJ (1000 times greater than the 1 kJ mean), measured by the very low frequency (VLF) ground stations of the World-Wide Lightning Location Network (WWLLN)^9–12^. Interestingly, the distribution of superbolt locations and occurrence times was not equivalent to that of ordinary lightning: instead, superbolts were found to occur over oceans and seas at a much higher rate, and more often in winter^7^. The north Atlantic (west of Europe) and the Mediterranean Sea have some of the highest wintertime occurrence rates of superbolts^7^ (see Fig. 1 and methods subsection Ground-based measurements of superbolts). As with any cloud-to-ground (CG) lightning flash, superbolts emit electromagnetic radiation in the very low frequency band that propagates within the Earth-ionosphere waveguide and escapes to the magnetosphere as whistler-mode waves along Earth’s magnetic field lines^13^.

Here, we show superbolt VLF electromagnetic (EM) power density in space. We combine space and ground-based measurements in a unique manner to follow electromagnetic superbolt signals from Earth to space over thousands of kilometers, to widely characterize their VLF electric and magnetic wave power density in space and on Earth, to compute ground-space transmitted power ratio, and to extract statistical electromagnetic properties of lightning superbolts never before reported. We conclude that, in addition to a location bias, superbolts exhibit other properties that differ from ordinary lightning, deepening the mystery associated with these extreme events.

## Results

### Superbolt electromagnetic power

**Fig. 2:**
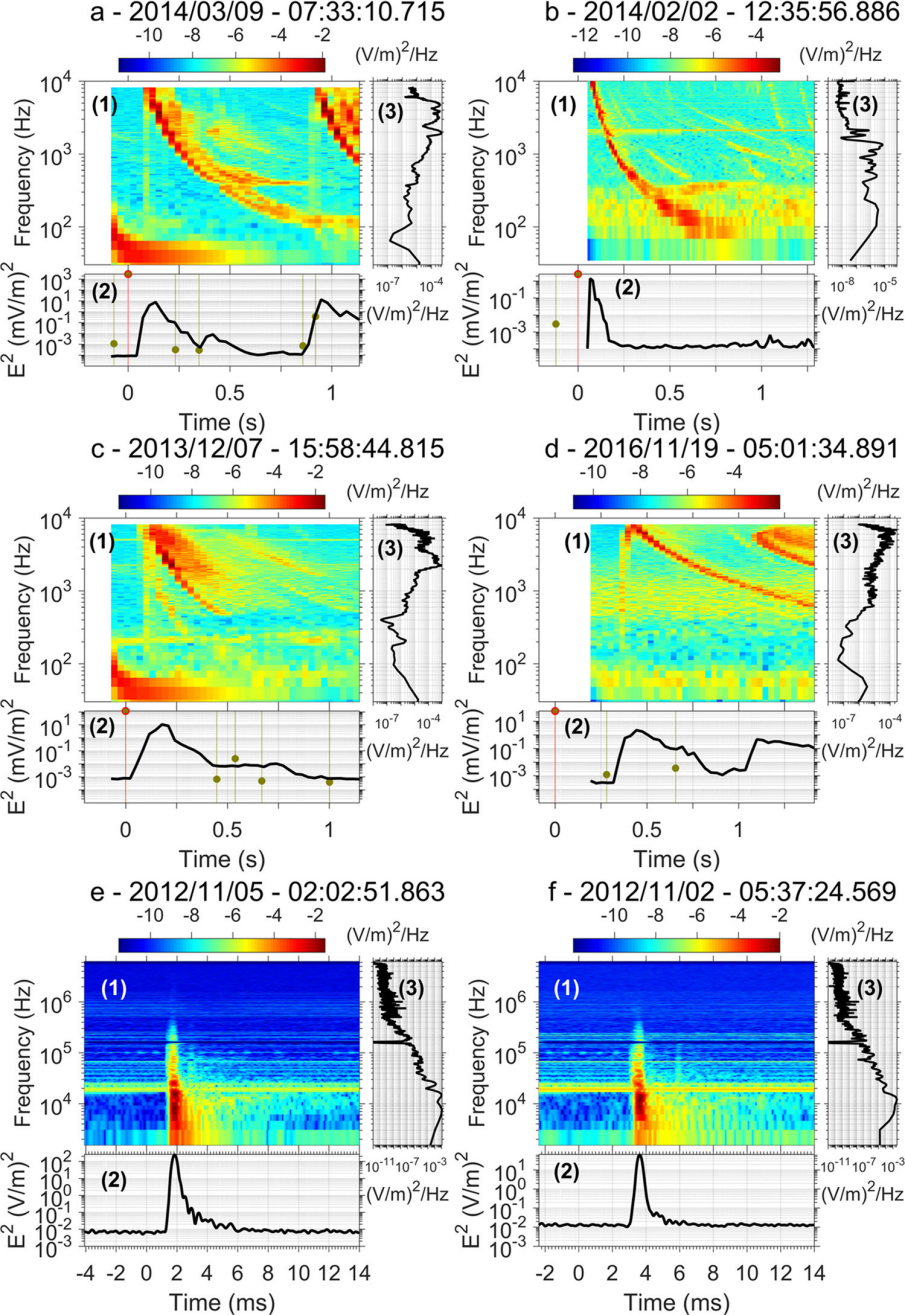
Superbolt’s electric power from space and ground-based measurements. **a**–**f** Show electric power spectral density and squared electric field (intensity) versus time for six different superbolts measured either (**a**–**d**) in burst mode in space (PSD in mV^2^/m^2^/Hz) or (**e**, **f**) on the ground (PSD in V^2^/m^2^/Hz). The specifics of the events are in Supplementary Tables 2 and 3. Panel a1, b1, c1, d1, e1, f1 show the evolving power spectral density. Panel a2, b2, c2, d2, e2, f2 show the evolving wave electric field intensity. Panel a3, b3, c3, d3, e3, f3 show the average of the PSD over 1 s (in V^2^/m^2^/Hz). Dashed vertical lines in the line plots (a2–f2) show times of all WWLLN-detected lightning during that time interval. Circles in the line plots (a2–f2) indicate an estimate of the median squared electric field in space based on WWLLN-measured lightning energy (computed from ref. ^22^). The non-superbolt median squared electric field estimates (green circles) are found to be >3 orders of magnitude less than the superbolt estimates (green circle with a red contour), confirming that the detected space-based signals correspond to the WWLLN superbolt, and are not caused by other lightning. Continuous signal at ~20 kHz (e1–f1) is due to a powerful VLF ground transmitter and should be disregarded. The sharp rising tone just prior to the main whistler profile in (a1, c1, d1), best seen in (d1) at *t* = 0.4 s for frequencies within 10^2^ and 10^4^ Hz, is an anti-aliasing filter effect with a fold over of the power above the top frequency.

**Fig. 3:**
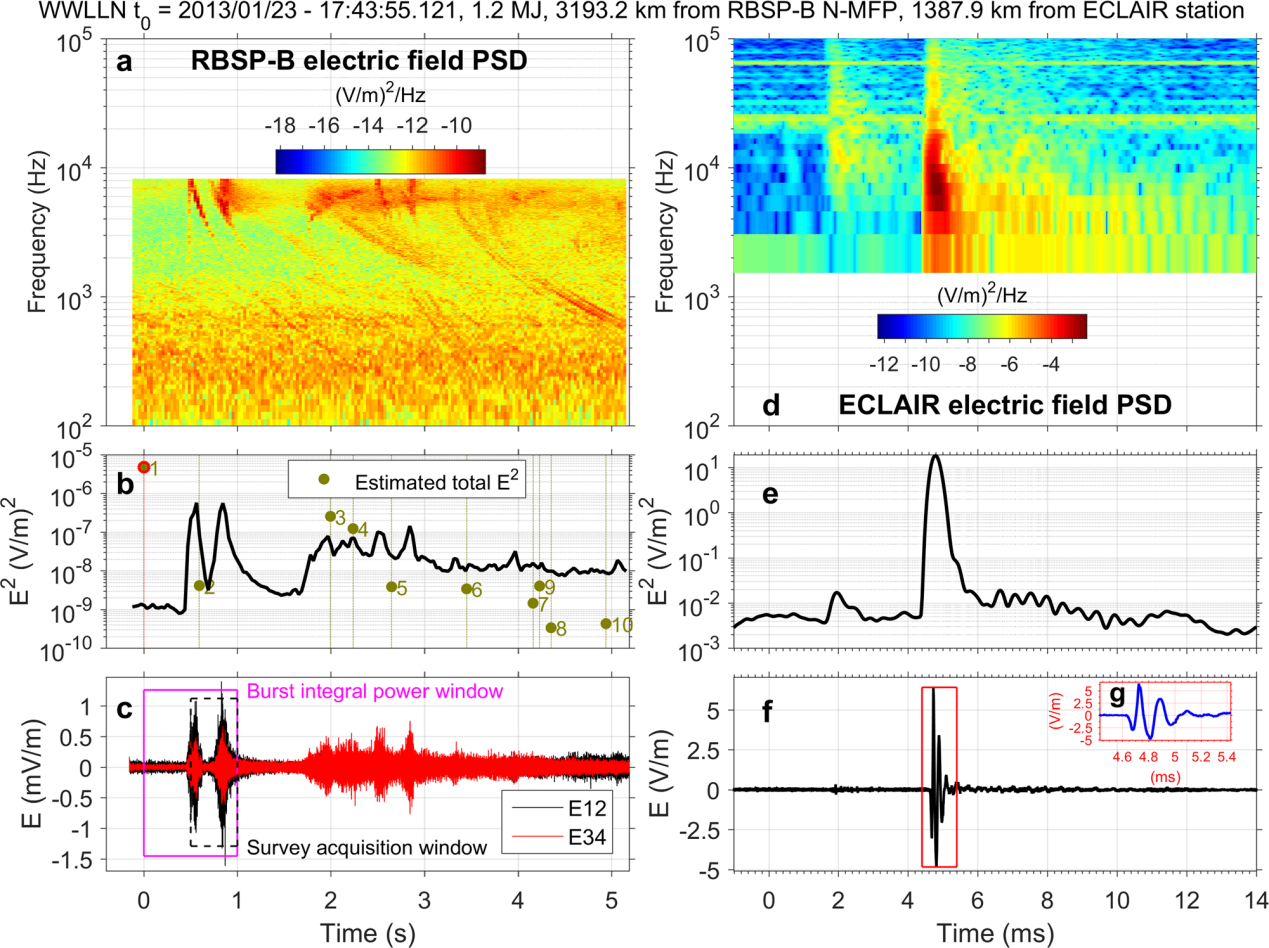
Simultaneous space and ground measurements of a superbolt. (Left) Space and (right) ground simultaneous measurements of a superbolt (1.2 MJ) detected by WWLLN with *t* = 0 at 2013/01/23-17.43.55.121 UTC. In space, we display the Van Allen Probes (RBSP) burst-mode measurements versus time of **a** the electric field power spectral density (PSD in V^2^/m^2^/Hz) measured by EFW, **b** the evolution of the squared electric field (intensity) and estimated time at the satellite of all WWLLN-detected lightning strokes in the time window (dashed vertical lines), and **c** the satellite-detected waveform with the survey acquisition and burst integration windows. (right) On the ground, we display (with same units) the evolution of **d** the electric field PSD in V^2^/m^2^/Hz and of **e** the squared electric field, **f** the electrical field waveform and **g** its zoom. The electric field PSD (**a**) has a characteristic descending tone shape in space (between *t* ~ 0.4–0.6 s) but shows a second wave (starting at *t* ~ 0.6 s) that is the bounce reflection of the primary wave. The sharp rising tone just prior to the descending tone shape (for frequencies within 2 × 10^2^ and 9 × 10^3^ Hz) is an anti-aliasing filter effect with a fold over of the power above the top frequency that should be disregarded. The estimated median squared electric field (in mV^2^/m^2^) (following^22^) of all other lightning is reported in **b** with green circles (#2–#10) and are found to be much lower than the superbolt’s intensity and its estimate (#1 green circle with a red contour) (more detail in Supplementary Table 4). The superbolt frequency decreases below 400 Hz (deep in the whistler-mode hiss wave band) after 2 s (**a**). See Supplementary Fig. 6 for more detail on space measurements and Supplementary Fig. 7 for more detail on ground-based measurements.

Superbolts are first located world-wide by making use of the WWLLN database, with an identification of superbolts from their energy greater than 1 MJ from synchronized measurements by WWLLN ground receivers^7^. In Europe, we make use of electric field measurements from a ground measurement campaign (ECLAIR)^14,15^ conducted by CEA as well as data recorded by the stations of the French lightning location network, Météorage (MTRG)^16^ (see methods subsection Ground-based measurements of superbolts). In space, we use two instruments^17,18^ on board the NASA Van Allen Probes mission^19^ to identify superbolt electric and magnetic wave fields (see methods subsection Space measurements of superbolts) from high-definition recordings. Superbolts signals are then gathered and retained after a selective screening process (see methods subsection Superbolt detection and selection). High-resolution electric power spectral density (PSD) from a selection of 6 superbolts is shown in Fig. 2, from space (a1–d1) and ground-based (e1–f1) measurements. The black line in Fig. 2 (a2–f2) is the wave electric (magnetic) intensity, *E*^2^ in V^2^/m^2^ (*B*^2^ in pT^2^), more commonly called, the wave power, which computation from the PSD is explained in the method section. In space, burst-mode spectrograms (Fig. 2 a1–d1) show superbolt VLF waves have a clear whistler-mode descending tone over a time period of seconds, as with all lightning-generated whistlers^20^, due to frequency-dependent dispersion through the ionosphere and plasmasphere. Their PSD frequency range spans ~0.1 to ~10 kHz, with most power above 2 kHz^21^ and within the first second. The sharp rising tone just prior to the descending tone shape (e.g., Fig. 2 d1 for frequencies within ~10^2^ and ~10^4^ Hz at *t* ~ 0.4) is an anti-aliasing filter that should be disregarded. Superbolts are observed in space with a time delay of 0.1 to 0.4 s from the superbolt WWLLN recorded time that marks *t* = 0. The delay corresponds to the time the wave takes to propagate away from the stroke location, along the Earth-ionosphere waveguide, and then through the ionosphere into the magnetosphere along field lines and ultimately to the satellite. In some cases, the wave propagates to the magnetically conjugate footprint and reflects back before being seen at the spacecraft (see discussion below related to the superbolt of Fig. 3). Event times of all WWLLN events in the satellite burst window are marked with dashed vertical lines in Fig. 2 (bottom line plots, with the superbolt time indicated in red and non-superbolt lightning signals in green). An empirical estimate of the median squared electric field at the satellite location is calculated and reported for each of these strokes^22,23^ based on both the estimated WWLLN total energy on the ground and the distance of the event (circles in the bottom line plots). The non-superbolt median squared electric field estimates (green circles) are lower than the superbolt estimates (red circle), by >3 orders of magnitude, confirming that the detected space-based signals only correspond to the WWLLN superbolt, and are not caused by other lightning.

### Simultaneous ground-based and space measurement of a superbolt

The closest of the two superbolts (1.2 MJ) that we observe synchronously from Earth and space is represented in Fig. 3 (more detail in Supplementary Fig. 6 and Supplementary Fig. 7). The waveforms (3c and 3f) are decomposed by Fourier transform to produce electric field (3a) and magnetic field (Supplementary Fig. 6e) PSDs. Other lightning activity (Fig. 3c) is composed of nine lightning events identified with WWLLN, listed in Supplementary Table 4, and plotted on a map in Supplementary Fig. 7f. Among these nine normal lightning events, event #3 is a strong 95.8 kJ stroke occurring ~2 s later. This lightning activity is also visible in space with whistler-mode waves (3a), showing good temporal alignment with the WWLLN signal and representing significant wave power (line plot in 3b and WWLLN estimated power) for times between 2 and 3 s. Nevertheless, this activity does not mask or perturb the superbolt burst-mode signal which persists for ~1 s of the first ~1.4 s of data. The superbolt signal is found to be composed of one main wave (with a 0.4 s delay from WWLLN detection time) and a second similarly powerful wave 0.2 s later (3a, 3c). The time it takes for the wave to travel from the satellite to the magnetically conjugate point along the field line at *L* ~ 2.43, reflect, and return at the equator is 0.2 s. Computing the Poynting flux (see Supplementary Method 1), we find a change of sign between these two waves, which indicates the second wave reaches the spacecraft from the opposite side of the field line. In the absence of any other event attested by WWLLN and since the time delay is consistent with one reflection, this suggests the two superbolt’s waves have entered the magnetosphere at different latitudes surrounding the source, followed then different paths, with one wave reaching first the spacecraft and the second reaching the spacecraft 0.2 s later after bouncing at the conjugate point of the field line. This is one of the plausible and classic scenarios obtained from ray-tracing simulations^24^. The peak squared electric field measured by an ECLAIR ground station for this event is 20 (V/m)^2^ 1387 km (3e) from the source. The average squared electric field over its duration (1.5 ms) is 4 (V/m)^2^, about 400 times greater than cloud-to-ground lightning measured on the ground (cf. discussion below). Quantifying the power spectral density on the ground is critical for validating ray-tracing simulations that predict the wave propagation from the source to space. The ground electric field peak (Fig. 3f and Supplementary Fig. 7e) is well resolved (at 0.08 µs from the 12.5 MHz acquisition) and presents a longer-than-average rise time (the time for the signal to go from 50% to 90% of peak magnitude), which is confirmed to be specific to superbolts from rise time and fall time statistics below. This leads to a somewhat more symmetric first peak. In space, the northern magnetic footprint of the Van Allen Probes North (3c) is 3193 km away from the superbolt location and the measured peak squared electric field is 6 × 10^−7^ (V/m)^2^, for a mean value of 8.5 × 10^−8^ (V/m)^2^ over 1 s, about 200 times higher than for normal lightning^21^. The root mean-squared (rms) amplitude of the magnetic field (3c), essential to compute pitch angle scattering of radiation belt electrons caused by lightning-generated whistlers in space (see review ref. ^25^), is 19 pT, 19 times larger than for normal lightning^21^. However, this is not as large as some of the superbolts we discuss next.

### Propagation and attenuation of superbolt electromagnetic signal

**Fig. 4:**
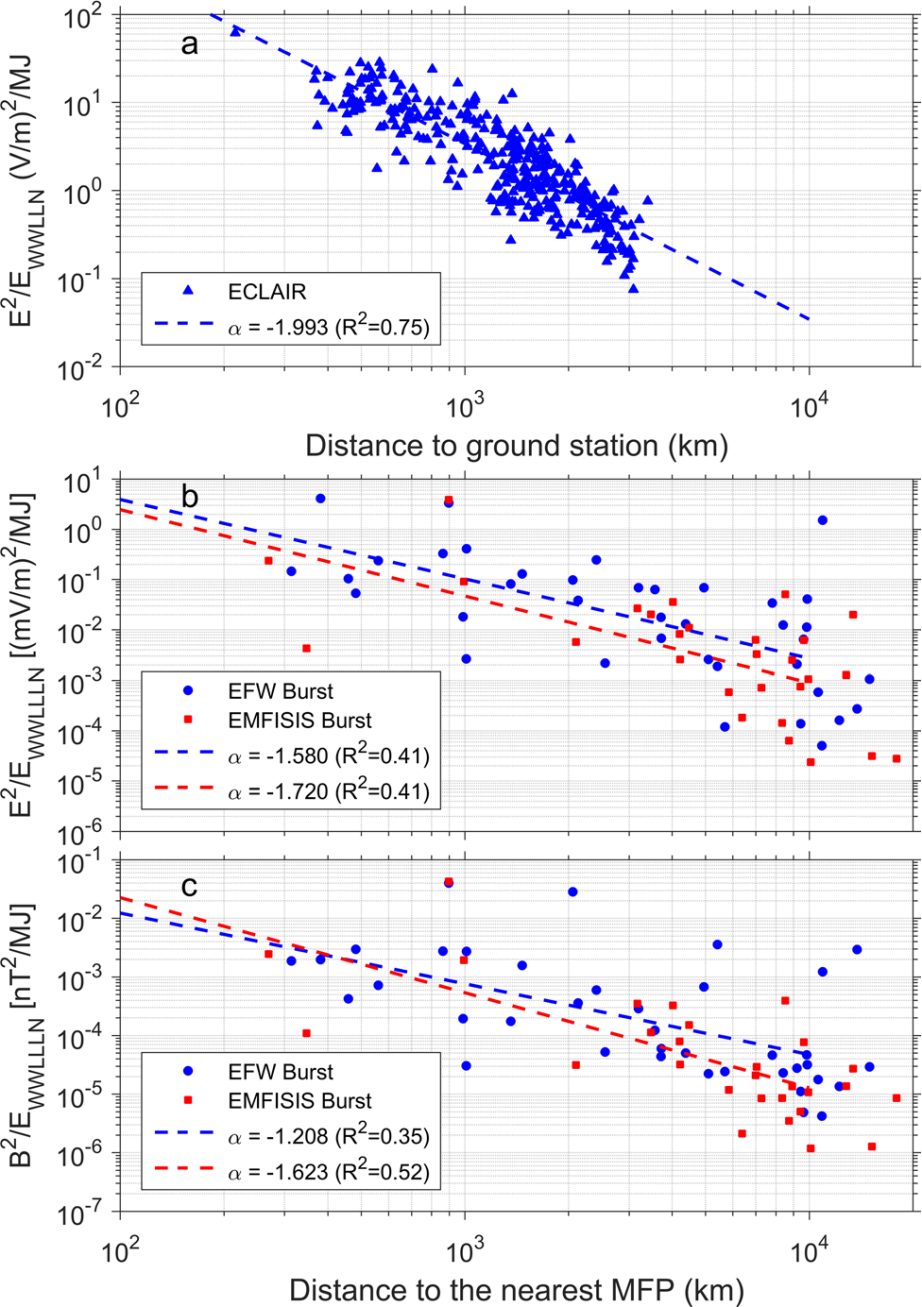
space and ground-based electric and magnetic power attenuation with distance. **a** Mean-squared electric field from 2 to 5 MHz of superbolts, averaged over 1.5 ms, and measured on the ground by ECLAIR and MTRG, then scaled by WWLLN stroke energy in MJ, as a function of distance from stroke location to ECLAIR and MTRG detecting stations. **b** Mean-squared electric field of superbolts in space in the VLF range, averaged over a 1 s duration, scaled by the WWLLN stroke energy, as a function of the distance from stroke location to the nearest magnetic footprint (MFP) of the Van Allen Probes, from (blue circle) EFW burst mode and (red square) EMFISIS burst mode. **c** Same as middle for the mean-squared magnetic field. Regressions give the decay by power laws (color indicates which type of measurements are used).

The method displayed in Fig. 3 (Supplementary Figs. 6 and 7) was applied to each of the 66 superbolts observed in burst mode in 2012–2018 (cf. list and characteristics in Supplementary Tables 5 and 6), to analyze electric and magnetic field magnitudes in space. Ground squared electric field and PSD is similarly extracted from the 368 ECLAIR ground measurements of superbolts^26^. Mean-squared electric and magnetic fields are computed over a time period starting at the WWLLN superbolt time and lasting 1.5 ms on the ground and 1 s in space. These means are then plotted in Fig. 4, scaled by the WWLLN energy, and plotted against distance from the source in order to present a unique global view of superbolts’ electromagnetic mean power density on Earth (Fig. 4a) and in Space (Fig. 4b, c). We find that ground EM power decay follows an inverse power law with a power ~2 over distance, which confirms a well-known far-field decay for EM waves in free space. In space, at the equator, superbolt EM power decays with an inverse power law with a power ~1.6 over distance. In comparison, normal lightning VLF power decays with a power law varying between ~1.7^27^ and ~2.3^23^ when measured from space at low altitude (~700 km) (see also ref. ^28^). VLF power loss between ground and space is expected due to wave power spreading out globally in the Earth-ionosphere waveguide and attenuation of the VLF signal by the atmosphere/ionosphere during propagation. The variability of power in space among individual superbolts with similar energy and distance is also found to be large (1–2 orders of magnitude). This variability is consistent with the great variability in L-shell, longitude, local time, and season of all lightning-generated waves^21^. However, such a variability limits the utility of the power density laws of Fig. 4 and put forward the need of reliable full modeling^29^.

### Ground-to-space transmission factor

Using these empirical fall-off relations with distance, we can rescale backward the space electric PSD and forward propagate the ground electric PSD observed in Fig. 3 to the same altitude (here 300 km where the law of Fig. 4b starts) and take their ratio to obtain a transmission factor from ground to space per frequency for the VLF range reported in Supplementary Table 7. The transmission factor is ~10^−8^ in the VLF range, which represents the lower bound of recent computations^29^, noticing the latter are widely spread over orders of magnitude according to simulation parameters. The transmission factor of the second event observed synchronously is also reported in Supplementary Table 7, smaller due to the far distance of the superbolt. These two unique and rare synchronous cases we report can serve for modeling the VLF wave emission by ray-tracing, starting from the source in the atmosphere and reaching the magnetic equator in space, which has important applications for radiation belt modeling^30^.

### Ground statistics of superbolts

**Fig. 5:**
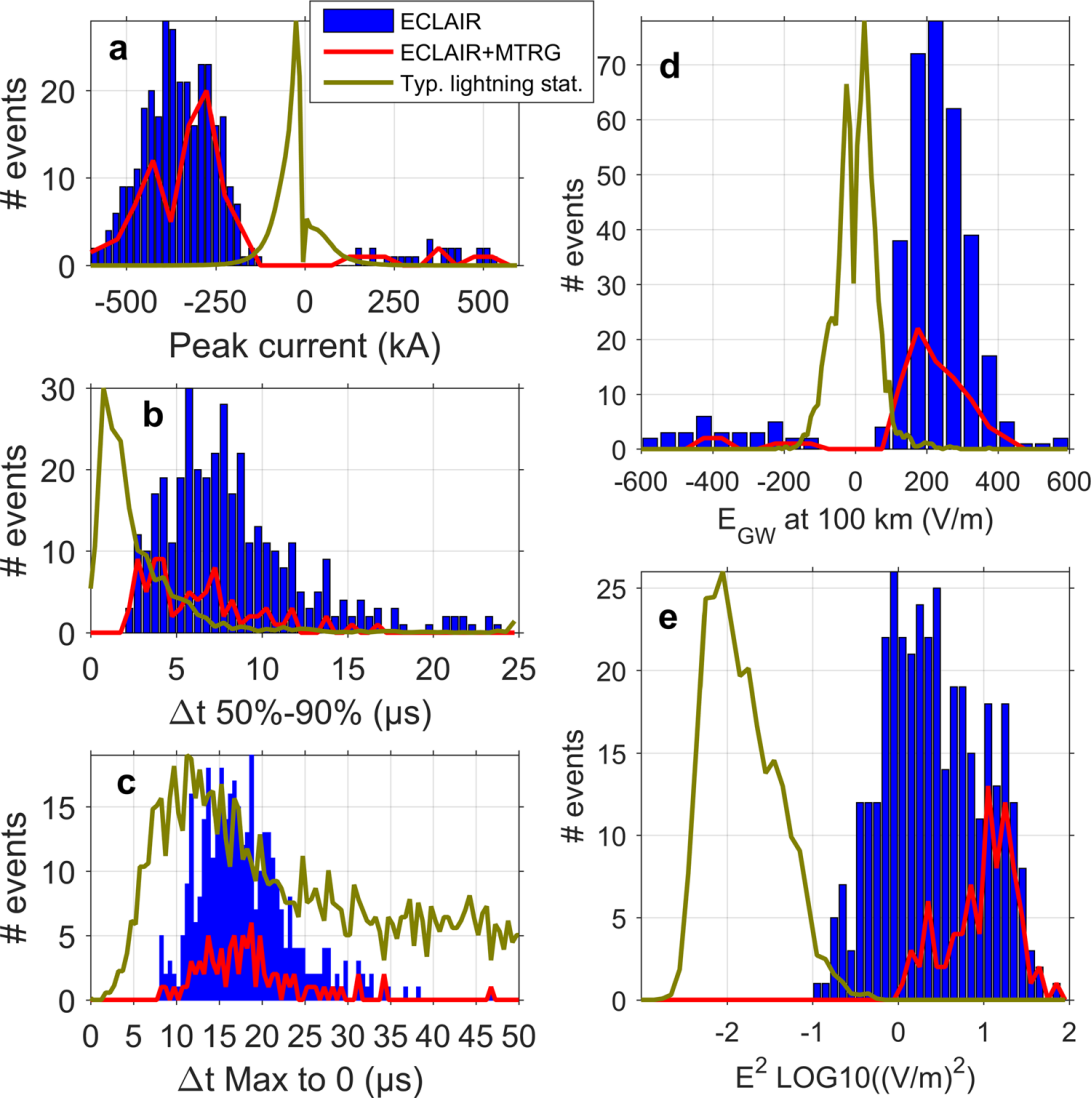
Ground-based measurements statistics related to the electromagnetic power of superbolts. Statistics of superbolt ground measurements from (blue) ECLAIR and (red) from both ECLAIR and MTRG stations compared with (green) the normalized statistics of regular lightning flashes measured by ECLAIR ground stations (from 3349 events and normalized to the maximum of superbolt statistics): **a** peak current estimate, **b** discharge rise time (time from 50 to 90% of peak), **c** discharge decay time, from maximum of the first peak to zero, **d** maximum electric field of the ground wave scaled to 100 km (using fits from Fig. 4), and **e** ground squared electric field, averaged over 1.5 ms. Notable findings are the symmetry of the superbolt’s discharge on the ground (**c**, **b**), peak current and electric field ~10 times larger than normal lightning (**a**, **d**), ground mean EM field squared about 100 times larger than for normal lightning (**e**).

Statistics of all superbolts measured with the ground network are plotted in Fig. 5a–e, along with statistics obtained from all 3349 lightning strokes measured from ECLAIR ground stations in the same period (09/2012-06/2013) (in green) in order to highlight the unique characteristics of superbolts (in red and blue). Superbolt median peak current estimate^31^ is 363 kA, ~10 times higher than normal lightning (panel 5a). Fifteen percent of superbolts are positive cloud-to-ground (+CG) flashes, compared with 28% of +CG lightning triggered during the whole campaign. While a typical rise time (Fig. 5b and Supplementary Fig. 8c) of normal lightning is 1–2 µs (up to 5 µs), superbolts last longer, 5–6 µs (up to 15 µs). The decay time of the ground wave (from maximum to zero) is between 50 and 90 µs for normal negative cloud-to-ground (-CG) lightning, giving a well-known asymmetric shape to the ground wave^5^ for normal strokes. On the contrary, superbolts have a fast decay time of 10–20 µs (panel 5c), comparable to the rise time (Fig. 5b and Supplementary Fig. 8c), leading to a more symmetric ground wave (as seen in Fig. 3f and Supplementary Fig. 7e). This remarkable symmetry of the discharge, existing, for instance, for narrow bipolar pulses^5^, could help reveal physical differences between superbolts and other lightning. The median of the ground wave electric field (reported at ground distance of 100 km) is +240 V/m to be compared with + /−20 V/m for normal lightning (panel 5d). Median squared electric field, computed over 1.5 ms is 2.6 (V/m)^2^ on the ground, about 100 times higher than for normal strokes (panel 5e). We find on average 38% of the total power (2 kHz–5 MHz) is in the VLF range (2–12 kHz) for normal lightning while this percentage reaches 68% for superbolts (cf. distribution in Supplementary Fig. 9a). This percentage increases as the lightning power increases (Supplementary Fig. 9b). This suggests that is the longer duration of powerful lightning that populates the lowest frequencies of the VLF range.

### Space statistics of superbolts

**Fig. 6:**
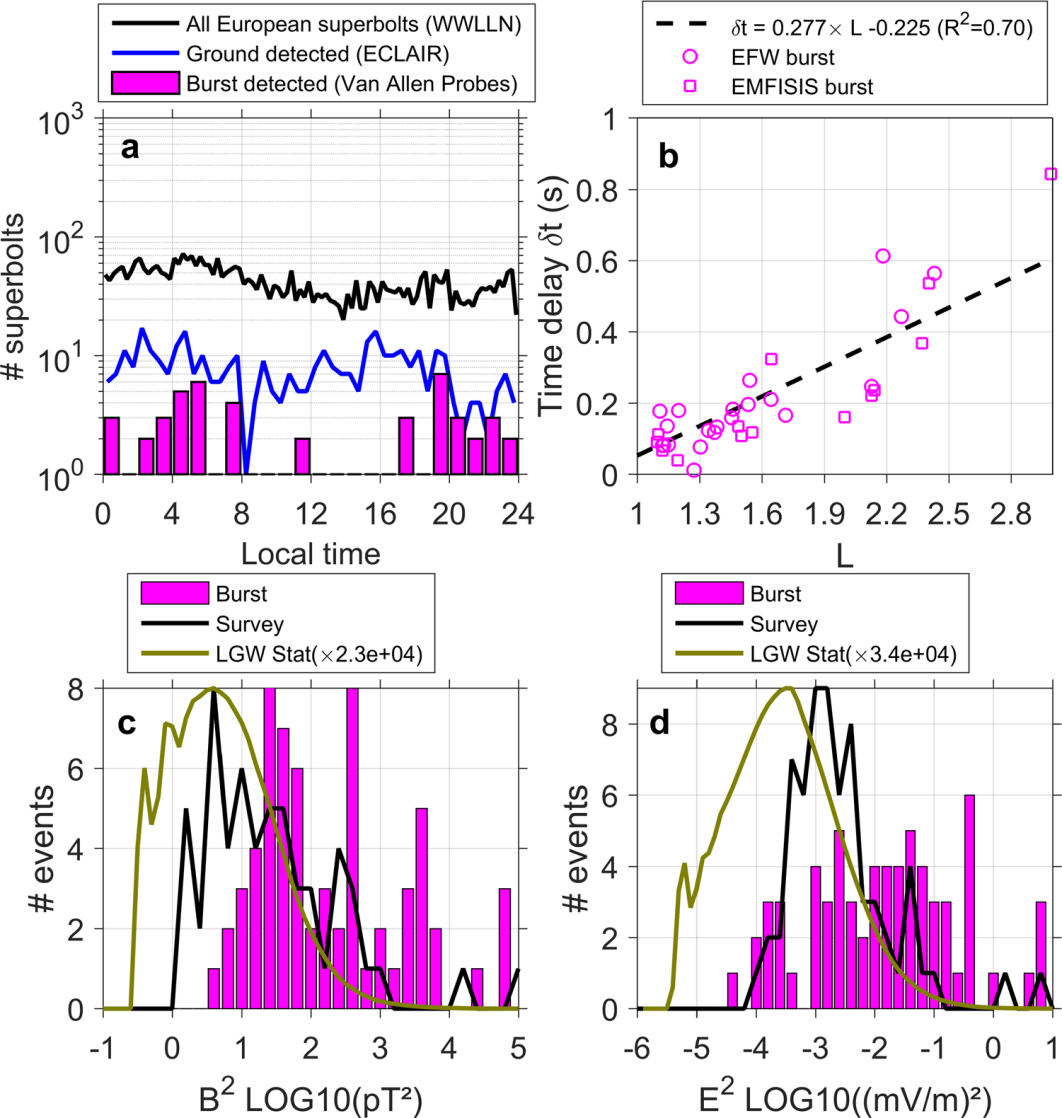
Space measurements statistics related to the electromagnetic power of superbolts. Superbolts’ local time (in hour) distribution (**a**) and **b** time delay from ground time to detection time in space versus L-shell (*t* = *αL* + *β*). Statistics of Van Allen Probes **c** magnetic and **d** electric field squared, averaged over 1 s, from (pink) burst and (black) survey-mode measurements compared with (green) regular VLF lightning-generated wave distributions^21^. The mean [median] of the magnetic (electric) field squared is 3598 pT^2^ (0.40 mV^2^/m^2^) [80 pT^2^ (0.015 mV^2^/m^2^)]. Notable findings are the absence of the superbolt’s electromagnetic signal in space during day times (**a**), a long-time delay of the space signal proportional to L-shell (**b**), ground and space mean EM field squared about 100 times larger than for normal lightning (**c**, **d**).

In space, another remarkable characteristic in these statistics is that superbolts are rare during day time, with 5/66 events occurring from ~9am to ~5 pm local time (Fig. 6a, pink histogram). Yet there is no sensitivity to day/night measured on the ground (6a, black/blue lines). This local time dependence is similar to the one found for non-superbolt lightning-generated waves (cf. second panel of Fig. 4 in ref. ^21^) and supports previous findings that the day time ionosphere makes the propagation of any lightning-generated waves into the magnetosphere more difficult. Superbolts detected in space from local burst-mode measurements are measured at a median satellite L-shell of 1.6 (~4000 km altitude at the equator). The mean [median] distance between WWLLN superbolt location and the closest magnetic footprint of the Van Allen Probes is 5924 km [5023 km]. They have a long-time delay (0.1–0.8 s) that corresponds to propagation time in the Earth-ionosphere waveguide and along field lines in the magnetosphere to the satellite, with some possible reflection between the conjugate points. The time delay, *δ*t, follows therefore a linear scaling with respect to L-shell, *δ*t = 0.277L – 0.225 in second, that is established in (panel 6b). Further ray-tracing studies should be able to answer whether this delay is due to *ducted* wave propagation along field lines in density depletions/enhancements (ducts) or to unducted wave propagation^32^, which is a general open problem of wave propagation^25,33^. Of the 10,724 superbolt events identified by WWLLN in 2012–2018, 431 WWLLN-detected superbolts were also captured by EMFISIS lower-resolution survey-mode data between 2012 and 2018. However, Van Allen Probes survey-mode measurements collect 0.5-s long records every 6 s. As superbolts signals in space typically exceed 1-s duration, the survey measurements usually do not contain the entire signal, thus truncating the total measured power. We show in Fig. 6(c, d) that survey-mode superbolt average power (black lines) is statistically larger than normal lightning, but not as powerful as burst-mode determined average power (pink histogram). This artificial bias is revealed by the burst-mode squared electric/magnetic field (pink, Fig. 6c/6d), which is found to be 10–1000 times more powerful than normal lightning (with global statistics^21^ scaled in green for comparison). The three strongest events have a ~250 pT (6 mV/m) wave magnetic (electric) rms amplitude in Fig. 6c, d. The mean magnetic (electric) rms amplitude of the 66 superbolts is 83 pT (873 µV/m) in Fig. 6c, d. In comparison, the mean magnetic (electric) rms amplitudes of lightning-generated waves computed from survey measurements of all types of lightning strokes (possibly including superbolts) is ~83 (~43) times lower, with 1 ± 1.6 pT (19 ± 59 μV/m)^21^.

The maximum of the burst-mode squared electric/magnetic field within the superbolt time window is provided in Supplementary Fig. 10. The comparison of Supplementary Fig. 10 with Fig. 6(c, d) shows the peak power exceeds by a factor ~10 (13) the mean magnetic (electric) power. Mean peak superbolt magnetic (electric) amplitude is 0.2 nT (1.9 mV^2^/m^2^) (Supplementary Fig. 10). Peak values are useful for nonlinear computations briefly mentioned below. Intercomparison of superbolt’s electromagnetic power with other natural waves such that whistler-mode hiss waves^20,34–42^, whistler-mode chorus waves^20,43^, and ElectroMagnetic Ion Cyclotron (EMIC) waves^44^ is left for future work.

## Discussion

Given spatial and temporal coverage of the Van Allen Probes data set, as well as the burst trigger algorithms, these events likely represent (to order-of-magnitude) the maximum power attainable for lightning-driven VLF waves near the Van Allen Probes. Such values may be useful for defining the dynamic range of future wave instruments or as an upper bound on lightning VLF wave power in simulations or numeric calculations. Extremely powerful lightning events including superbolts (>100 pT, representing 0.02% of lightning-generated waves) have been identified as strongly contributing to the global rms magnetic amplitude of lightning-generated waves for *L* < 2 (e.g., 44% at *L* = 1.1)^21^. These results suggest thus that the tail of the power density distribution of any type of waves (such as whistler-mode or EMIC waves) needs to be derived from, or at least confirmed by, from burst-mode measurements in order not to under-estimate the true wave power that is required for accurate radiation belt modeling (while today most statistical models are built from survey-mode data^21,34,35,37,39,41,42^). Finally, superbolt’s extreme power is a good candidate of highly powerful waves for which the quasilinear approximation (commonly used to compute wave-particle interactions in the radiation belts^45–50^) should break^51,52^, and to study further various nonlinear wave-particle interaction models^51–58^.

This study shows simultaneous measurements of a superbolt on the ground and the intense VLF signals in space driven by lightning. Ground and space observations demonstrate how different and extreme superbolts can be compared with normal lightning, for reasons not yet established. This study is a significant characterization of the previously unknown superbolts’ electromagnetic power, that should guide modeling and understanding of lightning electrodynamics, atmospheric discharges, and wave transmission from Earth to space, with applications in remote sensing, and wave modeling in space for radiation belt physics. Simultaneous optical and electromagnetic observations^59,60^ should be critical to help reveal more mysteries of superbolts.

## Methods

### Ground-based measurements of superbolts

The north Atlantic in western of Europe and the Mediterranean Sea in southern-western of Europe have some of the highest wintertime occurrence rates of superbolts^7^. This is a region covered by Commissariat à l’Energie Atomique et aux Energies Alternatives (CEA) ground stations^14,15^ in France. The ground stations measure electric fields from a few hundred Hertz to 5 MHz^14^ (VLF to High Frequency; HF) with vertical dipole whip antennas, at a sampling rate of 12.5 MHz.

The WWLLN network identifies 10,724 superbolts world-wide (gray dots, Supplementary Fig. 1) with a radiated energy greater than 1 MJ from synchronized measurements by >7 ground receivers^7^ (with a WWLLN residual time of group arrival for all stations of less than 30 μs) for the years 2012–2018, out of 1.5 × 10^9^ total strokes detected (see Supplementary Method 2 for the influence of the WWLLN minimum station number detecting a stroke and the residual time on the total number of superbolts). Thus, the occurrence frequency of superbolts in this WWLLN data set is seven events per million CG lightning events, yielding an average of 3.5 superbolt events per day world-wide (with a four times higher probability of occurrence in northern winter), and implying a very low probability to observe them simultaneously from space and on the ground. Among these 10,724 superbolts, 4034 were identified in Europe (Fig. 1), of which 384 occurred between 09/2012 and 06/2013, corresponding to a ground measurement campaign (ECLAIR) conducted by CEA. Of these, 368 high-resolution superbolt ground waveforms were recorded during ECLAIR^26^, with 86 of them also detected by the stations of the French lightning location network, Météorage (MTRG)^16^, within 1500 km of the ECLAIR network center (Fig. 1). These ground measurements (including WWLLN) provide superbolt occurrence time (with time accuracy <1 ms), geographic location, and WWLLN energy. They also provide ECLAIR high-resolution waveforms with rise and decay times, and ECLAIR and MTRG peak current estimates^31^ (see Supplementary Method 1 for more information).

### Space measurements of superbolts

In space, we use the Electric and Magnetic Field Instrument Suite and Integrated Science (EMFISIS) instrument suite^17^ and the Electric Fields and Waves (EFW) instrument^18^ on board the two probes (Probe A and Probe B) of the NASA Van Allen Probes spacecraft^19^ to measure electric and magnetic wave fields near the magnetic equatorial plane (+/−20° magnetic latitude). The instruments provide similar burst data, but capture records at different times. These ground and space-based measurements are used to study the symmetry of the first ground-wave peak, determine the time it takes for the signal to propagate to the satellite, quantify the loss of signal strength with distance, compute the frequency-dependent transmission of the wave power, and generate statistics (see Supplementary Method 1 for more information).

### Superbolt detection and selection

Of the 10,724 WWLLN superbolts, 1143 occurred during a time when Van Allen Probes EFW or EMFISIS were capturing burst data that records electromagnetic waves at high temporal resolution, called the waveform, although most of these waveforms did not measure a superbolt due to the satellite’s location. Only 212 of these burst captures occurred when the Van Allen Probes were at L-shell, L, less than three where most of lightning-generated waves are observed^21^. For facilitating further selection, composite figures (such as Figs. 2, 3 and Supplementary Figs. 6 and 7 discussed in the article) were generated for each of the 212 superbolts and used to screen them. Among them, 81 were retained because the on board burst system was triggered by identified whistler-mode waves and no unidentified (or other) radio wave power corrupted (partially or entirely) the burst signal. Additional selection criteria were then applied for retaining only clearly identified superbolts: (1) the lightning VLF signal was fully captured by the burst data (uninterrupted); (2) no other lightning flashes mask the superbolt signal (i.e., no lightning of higher or comparable estimated signal power^22,23^ at the satellite occurred within the burst window); (3) and the distance (direct line), *d*, from the superbolt location to the magnetic footpoint of the field line on which the satellite sits is <8000 km. Note that although we are requiring that the satellite *L* is less than three, there is no restriction on the superbolt location. For superbolts with *d* > 8000 km, we identified and retained some additional weak signals by hand that satisfied criteria (1) to (2). This process ensures the least possible contamination of the superbolt wave signal by other lightning. At the end of the selection process, only 66 burst captures of superbolt remained (38 from EFW and 28 from EMFISIS, with 1 recorded by both) out of the 1143 candidate events during which burst data was also collected. Among the 66 selected burst-mode signals, 33 were located by WWLLN to be over Europe (cf. Fig. 1), and only two of these were simultaneously recorded by ECLAIR, with one too far (9435 km away) from the closest Van Allen Probes magnetic footprint to be clearly identified. For these two events there is high-resolution data available from both the ground and space instrumentation that permits a detailed study linking the superbolt properties on the ground and in space, discussed below. Datasets are summarized in Supplementary Table 1.

### Superbolt electromagnetic power

High-resolution power spectral density (PSD) from a selection of six superbolts is shown in Fig. 2, computed from either the burst-mode electric field (a1–d1) or the ground-based (e1–f1) electric field waveforms. These waveforms (displayed in Supplementary Figs. 2, 3, 4) were decomposed by Fourier transform to produce electric field PSDs. The respective Van Allen Probes magnetic field PSDs are shown in Supplementary Fig. 5. Characteristics of all superbolts discussed specifically in the article are gathered in Supplementary Table 2 and 3. The black line in Fig. 2 (a2–f2) (see also the respective magnetic field intensity in Supplementary Fig. 5) represents the evolution of the square of the electric (magnetic) field wave, *E*^2^ in V^2^/m^2^ (*B*^2^ in pT^2^), a.k.a. the wave electric (magnetic) intensity or squared amplitude or, more commonly called, the wave power, that is the frequency integral of the electric (magnetic) field PSD over the VLF range in space (~2–10 kHz) and (~2 kHz–5 MHz) on the ground.

## Supplementary information

Supplementary Information

## Data Availability

ECLAIR ground waveforms^26^ are available at https://zenodo.org/record/3952425. Météorage data may be ordered from https://www.météorage.com. Van Allen Probes field data are available from the EFW and EMFISIS team websites, which one can link to here: http://rbspgway.jhuapl.edu. The WWLLN data are available from any WWLLN host or may be ordered from links (http://wwlln.net). The satellite Situation Center Locator operated online by NASA provides Van Allen Probes trajectories (https://sscweb.gsfc.nasa.gov/).

## References

[1] Turman BN (1977). Detection of lightning superbolts. J. Geophys. Res..

[2] Turman BN (1978). Analysis of lightning data from the DMSP satellite. J. Geophys. Res..

[3] Ripoll, J.-F., Zinn, J., Jeffery, C. A. & Colestock, P. L. On the dynamics of hot air plasmas related to lightning discharges: 1. Gas dynamics. *J. Geophys. Res. Atmos*. **119**, 10.1002/2013JD020067 (2014a).

[4] Ripoll J-F, Zinn J, Colestock PL, Jeffery CA (2014). On the dynamics of hot air plasmas related to lightning discharges: 2. *Electrodynamics*. J. Geophys. Res.: Atmos..

[5] Rakov, V. A. & Uman, M. A. *Lightning: Physics and Effects* (Cambridge Univ. Press, Cambridge, 2007).

[6] Borovsky JE (1998). Lightning energetics: Estimates of energy dissipation in channels, channel radii, and channel-heating risetimes. J. Geophys. Res..

[7] Holzworth RH, McCarthy MP, Brundell JB, Jacobson AR, Rodger CJ (2019). Global distribution of superbolts. J. Geophys. Res.: Atmos..

[8] Hutchins ML, Holzworth RH, Rodger CJ, Brundell JB (2012). Far-field power of lightning strokes as measured by the World Wide Lightning Location Network. J. Atmos. Ocean. Technol..

[9] Dowden RL, Brundell JB, Rodger CJ (2002). VLF lightning location by time of group arrival (TOGA) at multiple sites. J. Atmos. Sol.-Terrestrial Phys..

[10] Lay EH (2004). WWLL global lightning detection system: regional validation study in Brazil. Geophys. Res. Lett..

[11] Rodger C. J., Brundell, J. B., Holzworth, H. & Lay, E. H. *Growing Detection Efficiency of the World Wide Lightning Location Network, CP1118, Coupling of Thunderstorms and Lightning Discharges to Near-Earth* (eds Crosby, N. B., Huang, T. Y. & Rycroft, M. J.) (American Institute of Physics, 2009).

[12] Holzworth RH (2011). Lightning-generated whistler waves observed by probes on the Communication/Navigation Outage Forecast System satellite at low latitudes. J. Geophys. Res..

[13] Helliwell, R. A. *Whistlers and Related Ionospheric Phenomena* (Stanford University Press, 1965).

[14] Farges T, Blanc E (2011). Lightning and TLE electric fields and their impact on the ionosphere. C. R. Phys..

[15] Kolmašová I (2016). Subionospheric propagation and peak currents of preliminary breakdown pulses before negative cloud-to-ground lightning discharges. Geophys. Res. Lett..

[16] Pédeboy, S., Defer, E., & Schulz, W. *Performance of the EUCLID network in cloud lightning detection in the South-East France*. 8th HyMeX Workshop, Valletta, Malta (2014).

[17] Kletzing CA (2013). The electric and magnetic field instrument suite and integrated science (EMFISIS) on van allen probes. Space Sci. Rev..

[18] Wygant JR (2013). The electric field and waves instruments on the radiation belt storm probes mission. Space Sci. Rev..

[19] Mauk BH (2013). Science objectives and rationale for the radiation belt storm probes mission. Space Sci. Rev..

[20] Hospodarsky, G. B. et al. (2016). in Magnetosphere-ionosphere coupling in the solar system (eds Chappell, C. R. et al.) 127–143 (John Wiley, 2016).

[21] Ripoll J-F (2020). Analysis of electric and magnetic lightning-generated wave amplitudes measured by the Van Allen Probes. Geophys. Res. Lett..

[22] Burkholder BS, Hutchins ML, McCarthy MP, Pfaff RF, Holzworth RH (2013). Attenuation of lightning-produced sferics in the Earth-ionosphere waveguide and low-latitude ionosphere. J. Geophys. Res. Space Phys..

[23] Ripoll, J.-F., Farges, T., Lay, E. H., & Cunningham, G. S. (2019). Local and statistical maps of lightning-generated wave power density estimated at the Van Allen Probes footprints from the World-Wide Lightning Location Network database. *Geophys. Res. Lett*. **46**, 10.1029/2018GL081146.

[24] Bortnik, J. *Precipitation of Radiation Belt Electrons by Lightning-generated Magnetospherically Reflecting Whistlers*. Ph.D. thesis, Stanford University (2004). https://vlf.stanford.edu/pubs/theses

[25] Ripoll J-F (2020). Particle dynamics in the Earth’s radiation belts: review of current research and open questions. J. Geophys. Res..

[26] Farges, T. *ECLAIR Superbolt Waveforms*, 10.5281/zenodo.3952425 (2021).

[27] Fiser, J. C., Diendorfer, G., Parrot, M. & Santolik, O. Whistler intensities above thunderstorms. *Ann. Geophys.* **28**, 37–46 (2010).

[28] Jacobson AR, Holzworth RH, Pfaff RF, McCarthy MP (2011). Study of oblique whistlers in the low-latitude ionosphere, jointly with the C/NOFS satellite and the World-Wide Lightning Location Network. Ann. Geophys..

[29] Jacobson, A. R., Holzworth, R. H., Pfaff, R. & Heelis, R. Coordinated satellite observations of the very low frequency transmission through the ionospheric D layer at low latitudes, using broadband radio emissions from lightning. *J. Geophys. Res*. **123**. 10.1002/2017JA024942 (2018)

[30] Starks MJ, Albert JM, Ling A, O’Malley S, Quinn RA (2020). VLF transmitters and lightning-generated whistlers: 1. Modeling waves from source to space. J. Geophys. Res..

[31] Météorage. Calculation principle of peak intensity of stroke current. *Technical Note*, https://www.meteorage.com/sites/default/files/2018-12/TN%20-%20Calculation%20principle%20of%20peak%20intensity%20of%20stroke%20current_EN.pdf. (2015)

[32] Sonwalkar, V. S. In *Geospace Electromagnetic Waves and Radiation* (eds LaBelle, J. W. & Treumann, R. A.) Ch. 6, p. 141 (Springer, 2006) 10.1007/3-540-33203-0_6

[33] Clilverd MA (2008). Ground-based transmitter signals observed from space: ducted or nonducted?. J. Geophys. Res..

[34] Meredith NP (2018). Global model of plasmaspheric hiss from multiple satellite observations. J. Geophys. Res..

[35] Malaspina DM (2016). The distribution of plasmaspheric hiss wave power with respect to plasmapause location. Geophys. Res. Lett..

[36] Tsurutani BT, Falkowski BJ, Pickett JS, Santolik O, Lakhina GS (2015). Plasmaspheric hiss properties: observations from Polar. J. Geophys. Res..

[37] Hartley DP, Kletzing CA, Santolík O, Chen L, Horne RB (2018). Statistical properties of plasmaspheric hiss from Van Allen Probes observations. J. Geophys. Res..

[38] Gurnett DA (1995). The Polar plasma wave instrument. Space Sci. Rev..

[39] Falkowski BJ, Tsurutani BT, Lakhina GS, Pickett JS (2017). Two sources of dayside intense, quasi-coherent plasmaspheric hiss: a new mechanism for the slot region?. J. Geophys. Res..

[40] Tsurutani BT (2018). Plasmaspheric hiss: coherent and intense. J. Geophys. Res..

[41] Malaspina, D. M., Ripoll, J.-F., Chu, X., Hospodarsky, G. & Wygant, J. Variation in plasmaspheric hiss wave power with plasma density. *Geophys. Res. Lett*. **45**, 10.1029/2018GL078564 (2018)

[42] Shi, R. et al. Properties of whistler mode waves in Earth’s plasmasphere and plumes. *J. Geophys. Res*. **124**, 10.1029/2018JA026041 (2019)

[43] Cattell C (2008). Discovery of very large amplitude whistler- mode waves in Earth’s radiation belts. Geophys. Res. Lett..

[44] Engebretson MJ (2015). Van Allen probes, NOAA, GOES, and ground observations of an intense EMIC wave event extending over 12 h in magnetic local time. J. Geophys. Res..

[45] Abel B, Thorne RM (1998). Electron scattering and loss in Earth’s inner magnetosphere, 1: Dominant physical processes. J. Geophys. Res..

[46] Kim K-C, Shprits Y, Subbotin D, Ni B (2011). Understanding the dynamic evolution of the relativistic electron slot region including radial and pitch angle diffusion. J. Geophys. Res..

[47] Ripoll J-F, Chen Y, Fennell JF, Friedel RHW (2014). On long decays of electrons in the vicinity of the slot region observed by HEO3. J. Geophys. Res..

[48] Ripoll J-F (2017). Effects of whistler mode hiss waves in March 2013. J. Geophys. Res..

[49] Ripoll J‐F (2019). Observations and Fokker-Planck simulations of the L-shell, energy, and pitch angle structure of Earth’s electron radiation belts during quiet times. J. Geophys. Res..

[50] Zhao, H. et al. Plasmaspheric hiss waves generate a reversed energy spectrum of radiation belt electrons. *Nat. Phys*. 10.1038/s41567-018-0391-6 (2019).

[51] Albert JM, Bortnik J (2009). Nonlinear interaction of radiation belt electrons with electromagnetic ion cyclotron waves. Geophys. Res. Lett..

[52] Tao X, Bortnik J, Thorne RM, Albert JM, Li W (2012). Effects of amplitude modulation on nonlinear interactions between elec- trons and chorus waves. Geophys. Res. Lett..

[53] Nunn D, Omura Y (2015). A computational and theoretical investigation of nonlinear wave-particle interactions in oblique whistlers. J. Geophys. Res..

[54] Hsieh YK, Kubota Y, Omura Y (2020). Nonlinear evolution of radiation belt electron fluxes interacting with oblique whistler mode chorus emissions. J. Geophys. Res..

[55] da Silva CL (2018). Test-particle simulations of linear and nonlinear interactions between a 2-D whistler-mode wave packet and radiation belt electrons. Geophys. Res. Lett..

[56] Denton RE (2019). Pitch angle scattering of sub-MeV relativistic electrons by electromagnetic ion cyclotron waves. J. Geophys. Res..

[57] Tsurutani, B. T. et al. Low frequency (f<200 Hz) polar plasmaspherichiss: coherent and intense. *J. Geophys. Res.* **124**,10063–10084 (2019).

[58] Delzanno, G. L. & Roytershteyn, V. High-frequency plasma waves and pitch angle scattering induced by pulsed electron beams. *J. Geophys. Res*. **124**, 10.1029/2019JA027046 (2019).

[59] Lefeuvre, F. et al. TARANIS—a satellite project dedicated tothe physics of TLEs and TGFs. *Space Sci. Rev*. **137**, 301–315 (2008).

[60] Farges, T., Hébert, P., Le Mer-Dachard, F., Ravel, K. & Gaillac, S. MicroCameras and photometers (MCP) on board the TARANIS satellite, *XVI International Conference on Atmospheric Electricity*, 17–22 June, Nara, Japan (2018).

