# Supplementary material for: Electromagnetic power of lightning superbolts from Earth to space
Source: Nat Commun. 2021 Jun 11;12:3553. doi: 10.1038/s41467-021-23740-6 (PMC8196214; doi:10.1038/s41467-021-23740-6)
Supplement: Supplementary file 1 — Supplementary Information [file 41467_2021_23740_MOESM1_ESM.pdf]

SUPPLEMENTARY INFORMATION OF

Electromagnetic power of lightning superbolts from Earth to space

J.-F. Ripoll[1,2], T. Farges[1], D. M. Malaspina[3,4], G. S. Cunningham[5], E. H. Lay[5], G. B. Hospodarsky[6], C. A. Kletzing[6], J. R. Wygant[7], S. Pédeboy[8]

(1) CEA, DAM, DIF, F-91297 Arpajon, France
(2) UPS, CEA, 14 LMCE, 91680 Bruyères-le-Châtel, France
(3) Department of Astrophysical and Planetary Sciences, University of Colorado, Boulder, CO, USA
(4) Laboratory for Atmospheric and Space Physics, University of Colorado, Boulder, CO, USA
(5) Los Alamos National Laboratory, Los Alamos, New Mexico, USA
(6) Department of Physics and Astronomy, University of Iowa, Iowa City, IA, USA
(7) School of Physics and Astronomy, University of Minnesota, Minneapolis, Minnesota, USA
(8) Météorage, Pau, France

Contents of this file: Supplementary Method 1 and 2, Supplementary Tables 1, 2, 3, 4, 5, 6, 7, 8 and 9, Supplementary Figures 1, 2, 3, 4, 5, 6, 7, 8, 9, 10 and 11, and Supplementary References for a total of 20 pages.

Supplementary Method 1. More information about the measurements

On the ground

Each ECLAIR station is equipped with a vertical electric field antenna mounted on a mast, and a computer used for data digitalization and archiving[1,2]. The dipole antenna was designed and manufactured by CEA. Its pass-band is from 500 Hz to 5 MHz. The response inside this band is flat and was calibrated. The acquisition is achieved by comparison of the signal with a threshold of 1 V/m. The sampling frequency is 12.5 MHz, with a time window of 30 ms (including a pre-triggering time of 6 ms), a dynamic range of 14 bits, an idle time of less than 0.5 ms (between 2 triggering). Timing accuracy is ± 50 ns with GPS time-stamping. The spectrograms of ground data in Figure 2, Figure 3, and Supplementary Figure 6 were produced using 8172-point Fourier transforms with 95% overlap and a Hanning window.

In space

The twin Van Allen Probes spacecraft have an orbital perigee near 620 km altitude and an apogee near 5.8 Earth radii[3]. They are in near-equatorial orbits, sampling +/- 20 degrees magnetic latitude. Their orbital period is ~9 hours and their orbits precess through all local times every ~2 years. The spacecraft spin with a period of ~11 seconds.
This work uses survey and burst observations of VLF plasma waves from the EFW instrument[4] and the EMFISIS instrument suite[5] on the Van Allen Probes spacecraft. Both EFW and EMFISIS use the same sensors for data–collection - six voltage probes to measure electric fields and a three-axis search coil magnetometer (SCM) to measure wave magnetic fields.
The EMFISIS survey data used here consist of power spectral densities (PSD) calculated on-board the spacecraft using Fourier transforms of time-series E-field and B-field data. The survey data sample the first 0.5 seconds of each 6 seconds, and they consist of – 65 pseudo - logarithmically spaced frequency bins between ~2 Hz and ~11 kHz. In this analysis, the PSDs from all three axes of the SCM are summed, and the PSDs from the two

spin-plane electric field components are summed. The axial electric field component is not considered due to spacecraft noise concerns.

The EFW and EMFISIS burst data both consist of electric field signals on two orthogonal axes (in the spin plane) and three orthogonal axes of SCM data. The EFW burst data are sampled at 16,384 samples/s for ~5.5 second intervals, with a bandpass from ~100 Hz to ~8 kHz. The EMFISIS burst data are sampled at 35,000 sample/s for 6 second intervals with a bandpass from ~10 Hz to ~11 kHz. Some aliasing is observed when strong signals exist out of band (e.g. Figure 2d), creating vertical features in the burst spectrograms. The short intervals of EFW and EMFISIS burst data used here are selected on-board via triggering algorithms that search for intervals of high signal to noise. On the order of 70 burst intervals are collected, each, by EFW and by EMFISIS on a typical day. A fraction of these are collected at low L-shell (L < 3).

The spectrograms of Van Allen Probes burst data in Figure 2, Figure 3, Supplementary Figure 5, Supplementary Figure 6 were produced using 1024-point Fourier transforms with 50% overlap and a Hanning window[5]. Presented electric field PSD values are the sum of PSD values from the two electric field axes in the spacecraft spin plane. Presented SCM PSD values are the sum of PSD values from all three axes of SCM data.

In the section Simultaneous ground-based and space measurement of a superbolt of the main text, we calculate the wave Poynting flux direction by first rotating the burst time-series data into magnetic field-aligned coordinates. In this case, all three components of the search coil data are used, but only the two spacecraft spin-plane components of the electric field. This produces reasonable results because the ambient magnetic field is nearly in the spacecraft spin plane. Windowed fast Fourier transforms are performed, and the Poynting flux vector is calculated in the frequency domain such that, **S**, in each spectral bin, f, is $\mathbf{S}(f) = 1/\mu_0(\mathbf{E}(f) \times \mathbf{B}^*(f))$, with μ_0 the vacuum permeability, $\mathbf{B}^*$ is the complex conjugate of the Fourier transform of **B**, and **E** Fourier transform of **E**. The angle between the Poynting flux vector direction and the background magnetic field is calculated for each spectral bin to determine whether the VLF waves are moving along or against the local ambient magnetic field direction.

Supplementary Method 2. Influence of the WWLLN station number and residual time on superbolt statistics

This supplementary section discusses the difference between the number of WWLLN superbolts used in this article (10,724) and the number of WWLLN superbolts in the definitive WWLLN superbolt article[6]. There are two limiting criteria that most likely account for these different numbers: WWLLN residual value (goodness of fit) and the minimum number of WWLLN stations detecting a stroke. Both values are given in Supplementary Table 5 and 6 for each of the 66 superbolts.

The 'WWLLN residual' is the residual value in μs from the best fit minimization of time-of-group-arrivals (TOGAs) from all stations recording an event. This article used events with residuals < 30 μs. The work in [6] does not mention the residual value used to limit the data. We hypothesize, based on results shown below, that residuals < 25 μs were used in [6]. Increasing the residual time limit in this article increases tolerance and probability to measure a given event at the expense of location accuracy. However, the loss in location accuracy of less than 9 km for a 30 μs criteria is not significant in our study when those ground locations are mapped to the magnetosphere. The mean value of the WWLLN residual value of all superbolts in Supplementary Table 5 and 6 is 18.7 μs.

The minimum number of WWLLN stations detecting an event was set to eight for this article, while the minimum number required in [6] was seven. Increasing the minimum number of stations to eight in this paper adds confidence in the accuracy of the stroke energy determination[6], and minimally affects the total number of superbolts received. The mean value of the WWLLN station number of all superbolts in Supplementary Table 5 and 6 is 10.
Supplementary Table 8 shows the effect of minimum number of WWLLN receivers detecting the event and the WWLLN residual time on the total number of superbolts. These numbers are plotted in Supplementary Figure 10, showing the statistics of the number of superbolts in 2010-2019 for various residual times and its comparison with the statistics of [6] (their figure 9) referred in the main article. Values of the two studies agree within 10% on average, with 9290 superbols versus 10,724. In addition, we verify in Supplementary Table 9 that the number of superbolts we identify in space from the Van Allen Probes is not strongly dependent upon a WWLLN residual time of 25 versus 30 μs. Supplementary Table 9 shows that using a residual time of <25 μs instead of <30 μs would reduce the number of superbolts by 10% from 66 to 59 events.

Supplementary Tables

TYPE OF DATABASE MEASUREMENTS	DATABASE	Total number of lightning (world wide and including superbolts)	Total number of superbolt (in 01/2012-12/2018, world wide)	Selected number of superbolts (01/2012-12/2018, world wide)	Selected number of superbolts (01/2012-12/2018, Europe only)	Selected number of superbolts (09/2012-06/2013, Europe only)	Selected number of superbolts (09/2012-06/2013, Europe only) and in conjunction with ECLAIR ground measurements	Selected number of superbolts (09/2012-06/2013, Europe only) and in conjunction with ECLAIR and MTRG ground measurements
Ground-based	WWLLN	1.5E+09*	10 724	10 724	4034	384	368	86
Ground-based	ECLAIR	3349**	-	-	-	384	-	86
Ground-based	MTRG	-	-	-	-	-	86	-
Space	BURST DATA	1143+	212	66	30	2	2	0
Space	BURST DATA (EFW)	--	--	38	18	1	1	0
Space	BURST DATA (EMFISIS)	--	--	28	12	1	1	0
Space	SURVEY DATA	24E+06	431	431	139++	14	14	10
Space	SURVEY DATA (Electric)	--	--	--	--	10	10	5
Space	SURVEY DATA (Magnetic)	--	--	--	--	4	4	5

Supplementary Table 1: **Summary of datasets used in the article.** Ground and space-based datasets (from WWLLN, Van Allen Probes, ECLAIR ground stations, and Météorage) used in the study with counts of events and dataset overlap. (*) from 01/2012 to 12/2018. (**) Limited to 09/2012-06/2018 and to Europe. (+) Lightning-generated whistler or any other waves measured by EFW or EMFISIS burst mode. (++) Starting from 09/2012. (-) Non existing or not provided or irrelevant. (--) Data (Burst or survey) with electric field and magnetic field components not yet differentiated.

Superbolt date	WWLLN energy (MJ)	Superbolt latitude (°)	Superbolt longitude (°)	Distance (km) to Van Allen probes	Distance (km) to ground station	Van Allen Probes L-shell	Van Allen Probes MLT	Figure (#) or text
2013/12/07 15:58:44.815	2.012	-13.187	-169.584	1011	-	1.2	5.402	2a
2014/03/09 07:33:10.715	8.986	13.981	-105.708	482	-	1.14	0	2b
2016/11/19 05:01:34.891	2.353	49.84	-0.229	1467	-	2.27	4.499	2c
2014/02/02 12:35:56.886	1.144	38.94	-124.427	4020	-	1.12	1.758	2d
2012/11/02 05:37:24.573	1.86	55.184	7.286	-	1056	-	-	2e
2012/11/05 02:02:51.865	1.97	49.978	-1.232	-	522	-	-	2f
2013/01/23 17:43:55.121	1.225	42.035	19.244	3193	1387	2.43	21.47	3
2012/12/05 07:41:48.400	1.538	39.827	15.495	9435	1246	2.376	0.243	text

Supplementary Table 2: **Superbolts general information.** Information relative to the superbolts presented in Figure 2, Figure 3, and discussed in the text. See also Supplementary Table 3. Unavailable data is listed with a "-".

Superbolt date	E^2 (mV^2/m^2) (burst from space)	E^2 (mV^2/m^2) (at ground station)	B^2 (nT^2) (burst from space)	Peak current (kA)	Instrument (space and/or ground)	Figure (#) or text
2013/12/07 15:58:44.815	0.825	-	0.00549	-	EFW	2a
2014/03/09 07:33:10.715	0.4795	-	0.02645	-	EFW	2b
2016/11/19 05:01:34.891	0.303	-	0.00367	-	EFW	2c
2014/02/02 12:35:56.886	0.041	-	0.000371	-	EMFISIS	2d
2012/11/02 05:37:24.573	-	11.5E6	-	-367	CEA + MTRG	2e
2012/11/05 02:02:51.865	-	49.6E6	-	-414	CEA + MTRG	2f
2013/01/23 17:43:55.121	0.0843	3.63E6	0.00035	-326	EFW + CEA	3
2012/12/05 07:41:48.400	1.61E-4	1.27E6	7.7E-6	-191	EMFISIS + CEA	text

Supplementary Table 3: **Superbolts electromagnetic power.** Information relative to the superbolts presented in Figure 2, Figure 3, and discussed in the text. See also Supplementary Table 2. Unavailable data is listed with a "-".

#	Date	Time	Latitude (°)	Longitude (°)	Energy WWLLN (kJ)	Distance to South MFP (km)	Distance to North MFP (km)	Time in Figure 2 (s)	E^2 (Burkholder et al. 2013) $(mV/m)^2$
1	2013/01/23	17:43:55.121	42.04°N	19.24°E	1224.7	10475.9	3193.2	0.15	4.708e+00
2	2013/01/23	17:43:55.713	1.45°N	-9.11°E	10.6	9295.6	8463.4	0.75	4.116e-03
3	2013/01/23	17:43:57.117	42.42°N	10.49°E	95.8	10974.8	3744.2	2.15	2.533e-01
4	2013/01/23	17:43:57.362	-25.04°N	44.60°E	26.9	2967.1	8854.5	2.39	1.229e-01
5	2013/01/23	17:43:57.772	30.10°N	144.62°E	6.5	10812.7	7074.9	2.80	3.832e-03
6	2013/01/23	17:43:58.572	5.91°N	96.13°E	3.4	5648.4	6261.4	3.60	3.401e-03
7	2013/01/23	17:43:59.283	-3.63°N	104.32°E	1.2	5182.4	7614.7	4.32	1.440e-03
8	2013/01/23	17:43:59.477	-44.00°N	-50.82°E	1.0	9077.6	14992.1	4.51	3.358e-04
9	2013/01/23	17:43:59.351	36.90°N	11.87°E	1.8	10442.5	4044.9	4.38	4.021e-03
10	2013/01/23	17:44:00.062	-35.64°N	-68.03°E	1.9	10685.8	15512.9	5.10	4.242e-04

Supplementary Table 4: **Information about the superbol and the lightning events of Figure 3.** List and information of the superbolt (#1) of the 2013/01/23 shown in Figure 3 (and Supplementary Figure 6 and 7) and of all other lightning flashes (#2-9 in Figure 3 and Supplementary Figure 6 and 7) recorded by WWLLN during the superbolt burst signal, with their estimated squared electric field (last row) reported in Figure 3b and Supplementary Figure 6c. The estimated squared electric field is computed by combining the WWLLN total energy (itself determined over a 1.33 ms in[7]) of each lightning stroke and the stroke's distance in the empirical law extracted from the Figure 1 of [8], given by: $E^2(mV^2/m^2)/E_{WWLLN}(kJ)=10^{(-2.35\ \log10(d(km))+5.8197)}$. Note that Figure 1 of [8] is based on measurements from the Communications/Navigation Outage Forecasting System (C/NOFS) satellite orbiting at an altitude of ~700 km. $E^2(mV^2/m^2)$ is a median of the C/NOFS squared amplitude, with error bars of ~1 order of magnitude for distances below than 7000 km and larger above. $E^2(mV^2/m^2)$ is produced by the integration of the PSD in the VLF range, from 6 to 16 kHz, therefore a slightly different range than in this study. Worldwide WWLLN detection efficiency is about 10% on average, but rises to 50-70% of strong lightning[9,10], which are the events focused on in this superbolt study.

#	DATE & TIME	Latitude (°)	Longitude (°)	Distance (km)	L	nb stations	Residue (μs)	Energy (kJ)
1	2013/10/29 00:00:14.665	51	1.7	2554.6	1.31	10	16.1	1181.4
2	2013/11/24 04:43:05.015	42.5	18	2132.1	1.12	17	18.5	1064.4
3	2013/11/27 04:42:48.149	36.6	32.3	988.7	1.37	10	11.2	1129.9
4	2013/12/24 20:15:29.613	52.6	-14.9	313.9	2.18	14	23	2532.9
5	2016/12/14 05:35:15.835	35.4	36.1	2059.6	1.54	8	8	1856.5
6	2013/01/23 17:43:55.121	42	19.2	3193.2	2.43	12	11.2	1224.7
7	2013/07/05 04:38:01.112	-38.3	-7.8	458.7	1.71	9	17.5	1152.6
8	2013/11/25 21:14:48.720	38.9	18.7	561.2	1.64	14	17.9	1673.4
9	2013/11/26 06:51:48.884	38.3	21.4	1010.5	1.38	10	19.8	1543.5
10	2013/12/13 05:11:23.739	33.8	33.9	864.2	1.53	12	22.3	1128.6
11	2013/12/15 19:19:57.141	-14.5	-69.8	8401.2	2.5	10	24.2	1364.6
12	2016/02/28 02:27:44.353	-6.7	12.3	3563.5	1.98	8	14.1	1107.7
13	2017/01/14 00:51:18.848	-16.4	-69.9	10950.1	1.98	8	24.9	4192.3
14	2017/01/18 04:40:09.731	37.2	7.3	381.4	1.45	11	20.2	1321.1
15	2013/12/07 15:58:44.815	-13.2	-169.6	1011.3	1.2	14	26.5	2012
16	2014/02/16 00:05:40.113	-13.6	-72.4	14965.8	1.58	12	19.9	1835.2
17	2014/03/29 21:43:01.250	25.7	107.8	2408.2	1.82	8	14.3	1553.3
18	2015/04/06 01:24:28.927	-15.8	-65.5	13768.5	1.32	9	14.2	1230.9
19	2013/11/21 08:39:02.426	42.1	19.5	9629.1	2.27	10	19.1	5590.6
20	2013/11/24 00:28:44.969	9.9	-37.4	9832.3	1.11	9	20.9	1044.9
21	2013/12/26 18:01:43.568	37.2	9.7	12229.6	1.23	18	23.6	1822.8
22	2013/09/25 16:38:27.467	-40.4	114.3	898	2.13	8	29.1	1349
23	2013/11/26 09:43:44.460	41.8	-141.7	4382.3	2.82	9	23.5	1033.9
24	2013/11/28 16:12:35.466	11.8	93.9	3719.9	1.27	9	20.9	1557
25	2014/01/19 16:26:30.545	39.9	-162.1	3710.5	1.13	11	20.2	1352
26	2017/03/20 19:58:30.033	36.2	177.3	5415.2	1.33	9	17.4	1126.6
27	2013/11/22 21:25:29.911	40.1	8.2	9216.3	1.12	8	20.3	1012
28	2013/12/02 15:59:29.882	39.5	17.4	10619	1.15	8	10	1564
29	2013/12/27 11:17:38.044	36	12.8	7808.6	2.38	9	18.9	1398.1
30	2013/12/27 11:20:06.321	-34.1	5.5	9870	2.27	9	9.8	1080.3
31	2014/02/05 17:59:57.118	-8.1	178.8	5687.8	1.13	8	16.5	1047.8
32	2014/03/09 07:33:10.715	14	-105.7	482.3	1.15	8	13.7	8986.1
33	2013/11/23 06:12:34.388	41.7	12.3	9451.1	1.3	9	17.1	2199.7
34	2013/12/03 00:49:50.336	39.6	16.7	4947.5	1.46	8	11.7	2805.7
35	2017/03/18 09:45:51.613	-5.6	150.2	10891.7	1.34	9	14.6	5902.5
36	2013/10/26 09:42:33.732	40.8	-173.2	1361.1	2.36	8	12.7	2061.2
37	2013/12/02 15:55:41.372	-7.6	172.8	5099.2	1.24	9	21	1921.9
38	2016/11/19 05:01:34.891	49.8	-0.2	1466.8	2.27	12	17.3	2353.4

Supplementary Table 5: **Superbolts measured with EFW.** List and properties of the 38 superbolts identified with WWLLN (by more than 7 stations and with a residue lower than 30 μs) and measured in space with the EFW instrument.

#	DATE	TIME	Latitude (°)	Longitude (°)	Distance (km)	L	nb stations	Residue (μs)	Energy (kJ)
1	15/01/2015	07:41:48.400	-9.9	-74.6	17937.3	1.12	9	10.3	1545.9
2	07/10/2014	18:15:31.556	50.1	-13.6	5843.8	2	8	11.8	1913.4
3	11/03/2016	11:10:21.819	36.4	2.4	2103.8	2.42	8	12.8	1216.4
4	25/11/2013	02:13:00.266	39.9	13.4	8343.8	1.84	14	13.5	1345.4
5	18/01/2017	16:38:27.467	37.4	6.7	9640.8	1.98	12	13.6	1920
6	31/07/2014	22:37:08.699	-55.8	171	4203.8	1.2	9	15.1	1001.5
7	18/12/2017	21:24:57.153	-30.3	137.5	10095.6	2.25	8	15.1	4046.7
8	05/12/2012	10:07:28.665	39.8	15.5	9435.2	2.38	9	16.1	1538.4
9	20/01/2015	02:17:10.916	-17.9	173.3	8731.9	1.78	11	16.7	3086.8
10	10/12/2014	12:35:56.886	59.1	-5.9	993.5	2.18	13	17.2	1080.4
11	24/11/2013	19:14:15.103	37.3	7.3	6986.7	1.1	8	17.7	1183.6
12	28/02/2016	08:08:35.067	42	7.2	269.7	1.5	15	18.4	1458.8
13	25/11/2017	12:00:31.628	-5.8	163.8	4216.7	1.65	9	18.4	20796.6
14	28/12/2014	17:18:11.460	-13.2	-73	13406.1	2.18	8	18.4	2997
15	20/12/2013	01:52:10.705	60.6	4.9	347.1	2.99	13	18.6	3008.4
16	15/12/2013	08:25:53.373	57.7	-17.2	8920.2	1.1	18	19.1	1217.2
17	11/06/2016	21:17:21.090	-32.7	-26.6	6389.6	1.69	10	22.1	4494.8
18	28/04/2014	03:04:34.921	-1.7	140.3	15217.2	1.67	8	22.3	4157.3
19	31/07/2013	03:13:45.478	12.1	-16.7	7272.4	2.4	9	23	4192.4
20	30/07/2013	20:01:23.245	29.8	100.4	12820	1.48	12	23.3	1229.4
21	16/01/2016	02:06:36.557	38.3	6.1	8527.4	1.55	10	23.3	1028.2
22	14/09/2013	03:50:48.326	41.1	-107.2	4483.6	2.14	9	24.1	1777.9
23	02/02/2014	02:21:17.640	38.9	-124.4	4020.3	1.12	8	24.5	1144.3
24	17/05/2013	10:41:12.228	-9	168.2	3172.1	1.84	12	26.4	1004.8
25	14/11/2015	04:21:33.370	54.3	9.7	3474.5	1.49	13	26.7	1268
26	27/02/2016	04:52:10.841	36.7	1.7	9940.3	2.37	9	27.5	1438.2
27	25/09/2013	05:24:10.512	-40.4	114.3	898	2.13	8	29.1	1349
28	05/01/2014	17:18:08.898	12.6	-45.7	7038.5	1.12	10	29.6	1387.6

Supplementary Table 6: **Superbolts measured with EMFISIS.** List and properties of the 28 superbolts identified with WWLLN (by more than 7 stations and with a residue lower than 30 μs) and measured in space with the EMFISIS instrument.

23/01/2013 17:43:55 UT			
Frequence (Hz)	PSD (ground)	PSD (EFW, burst)	Transmission factor
1523	1.15E-05	1.83E-07	3.30E-08
3054	6.92E-05	2.53E-07	7.52E-09
4577	5.07E-04	9.47E-06	3.85E-08
6100	9.49E-04	1.82E-05	3.95E-08
7631	6.38E-04	1.01E-05	3.27E-08
8674	2.18E-04	3.63E-06	3.43E-08
3054-8674	3.63	8.43E-02	4.8E-8
23/01/2013 17:43:55 UT			
Frequence (Hz)	PSD (ground)	PSD (EMFISIS, survey)	Transmission factor
3110	6.92E-05	2.71E-07	8.08E-09
4526	5.07E-04	1.57E-05	6.38E-08
6212	9.49E-04	1.75E-05	3.80E-08
7793	6.38E-04	1.07E-05	3.46E-08
9041	2.18E-04	2.27E-06	2.15E-08
10351	7.97E-05	1.44E-07	3.73E-09
11724	5.21E-05	1.54E-08	6.10E-10
05/12/2012 07:41:48 UT			
Frequence (Hz)	PSD (ground)	PSD (EMFISIS burst)	Transmission factor
1532	1.34E-06	7.92E-09	8.52E-08
3047	1.75E-06	1.24E-08	1.02E-07
4579	2.84E-05	2.01E-08	1.02E-08
6111	1.14E-04	1.21E-08	1.53E-09
7626	2.15E-04	5.62E-09	3.78E-10
9141	2.63E-04	2.00E-09	1.10E-10
10690	2.10E-04	2.69E-09	1.85E-10
12205	1.29E-04	8.26E-10	9.22E-11
3047-12205	1.27	1.60E-04	1.80E-09

Supplementary Table 7: **Superbolt transmission factors**. Transmission factors of the two superbolts observed simultaneously on Earth and in space. Transmission factor computed at a given frequency from the ratio of the space PSD (averaged over 1 s) with the ground PSD (averaged over 1.5 ms) both in $mV^2/m^2/Hz$ scaled at 300 km (using laws of Figure 4) for both superbolts, which are observed synchronously on Earth and in space on the 2013/01/23 (cf. Figure 3 and Supplementary Figure 6 and Supplementary Table 2) and on the 2012/12/05. On the 2013/01/23, both survey and burst time windows turned out to be perfectly synchronized (cf. Figure 2c in the main text). This allows to use safely the survey data and to verify that both methods, which are very different from each other (survey PSD are directly computed on board, as explained in Supplementary Method 1, while burst PSD are computed by the authors as explained in the text), lead to similar PSD and, thus, transmission factors. Last line reports the PSD integrated over the written frequency range, in mV^2/m^2, and the associated transmission factor.

Number of Stations	Residue (in µs)	Total number of events in 2012-2018	Events in 2012-2016	Events in 2016-2018
7	10	1425	1091	334
7	15	5343	4293	1050
7	20	10258	8432	1826
7	25	14067	11613	2454
7	30	16647	13722	2925
8	10	573	444	129
8	15	2819	2318	501
8	20	6240	5253	987
8	25	8958	7609	1349
8	30	10724	9100	1624

Supplementary Table 8: **Influence of the number of stations and residual time.** Number of localized superbolts in (third column) 2012-2018, (fourth column) 2012-2016, and (fifth column) 2016-2018 according to the minimum number of VLF ground stations detecting an event (first column), and residual time limit (second column).

Residue (in µs)	EFW (# events)	EMFISIS (# events)	EFW+EMFISIS (# events)
<10	2	0	2
<15	10	5	15
<20	24	16	40
<25	36	23	59
<30	38	28	66

Supplementary Table 9: **Influence of the WWLLN residual time.** Number of superbolts localized by WWLLN for 8 stations and a given (first column) residual time identified in space from (second column) EFW, (third column) EMFISIS, (fourth column) both EFW and EMFISIS of the NASA Van Allen Probes.

Supplementary Figures

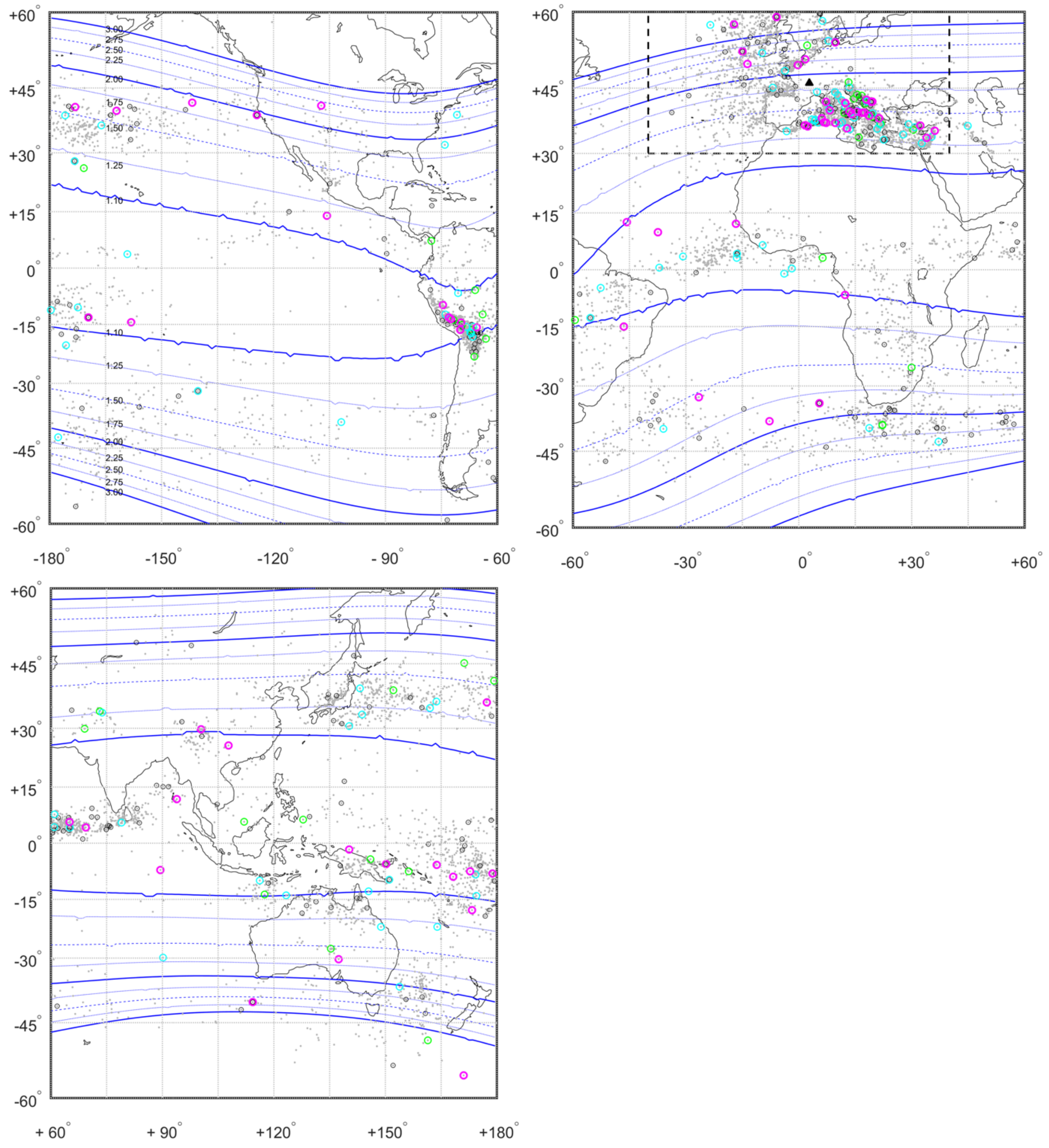

Supplementary Figure 1: **World-wide localization of the superbolts of this study.** The map is cut in 3 pieces of 120° longitude each and superbolts are shown with dots and symbols. (grey dots) WWLLN-detected superbolt (>1 MJ) in 01/01/2012-31/12/2018 (10 724 events), (black circles) coincident Van Allen Probes survey data (431 events), total burst data from (green circles) EFW (83 events) and (cyan circles) EMFISIS (129 events) before selection, and (pink circles) selected burst data from EFW and EMFISIS used in the study (66 events). IGRF L-shell from L=1.1 to L=3 (blue line) lines are projected at 100 km of altitude. Each L-shell line is labelled on the left of the map: (plain lines) L=1.1, 2., 3, (dotted lines) L=1.25, 1.75, 2.25, 2.75, (dashed lines) L=1.5, 2.5. The box (black dashed-line) indicates the geographical region covered by Figure 1. The black triangle indicates the location of one of the ECLAIR stations used in the article. The map itself is made with ©Matlab Mapping Toolbox.

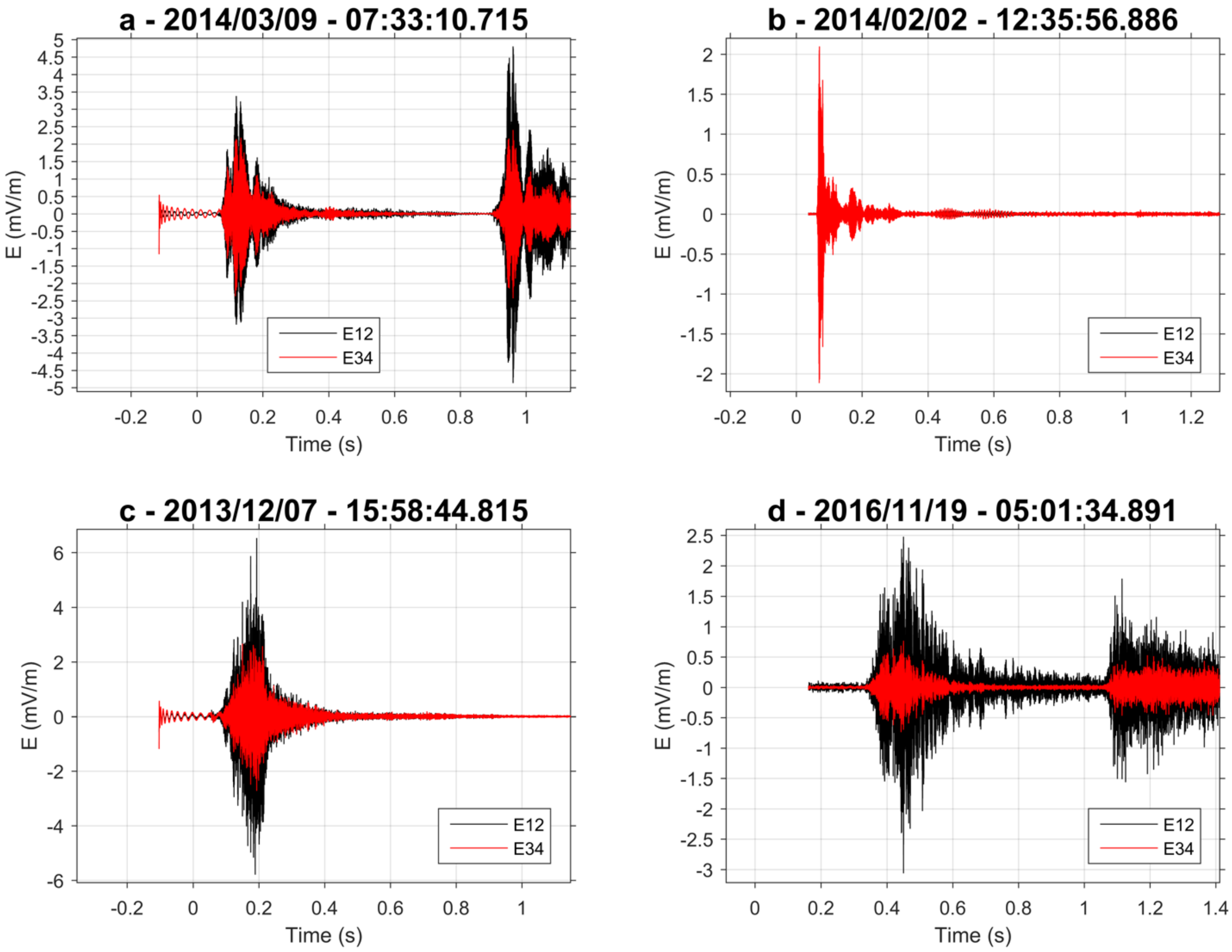

Supplementary Figure 2: **Superbolt electric field waveforms**. electric field waveform of the superbolts electric field waveform of the superbolts measured in space and presented in Figure 2 of the main text. The waveforms are used to compute the electric field PSDs. (a-d) waveforms correspond to (a-d) PSDs of Figure 2 of the main text.

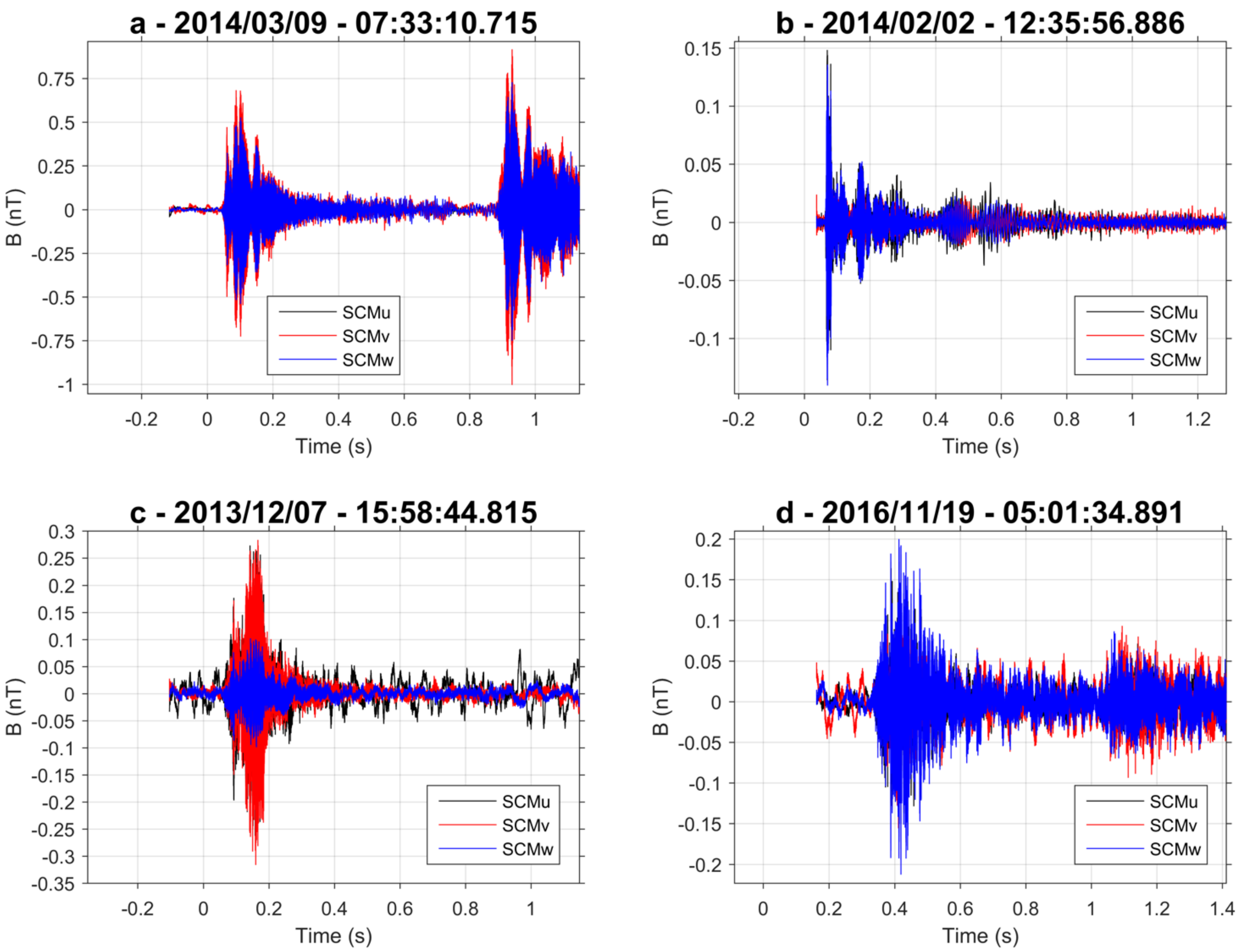

Supplementary Figure 3: **Superbolt magnetic field waveforms**. magnetic field waveform of the superbolts measured in space and presented in Figure 2 of the main text and Supplementary Figure 5. The magnetic field waveforms are used to compute the magnetic field PSDs, which is plotted in Supplementary Figure 5. Superbolts are plotted in the same order.

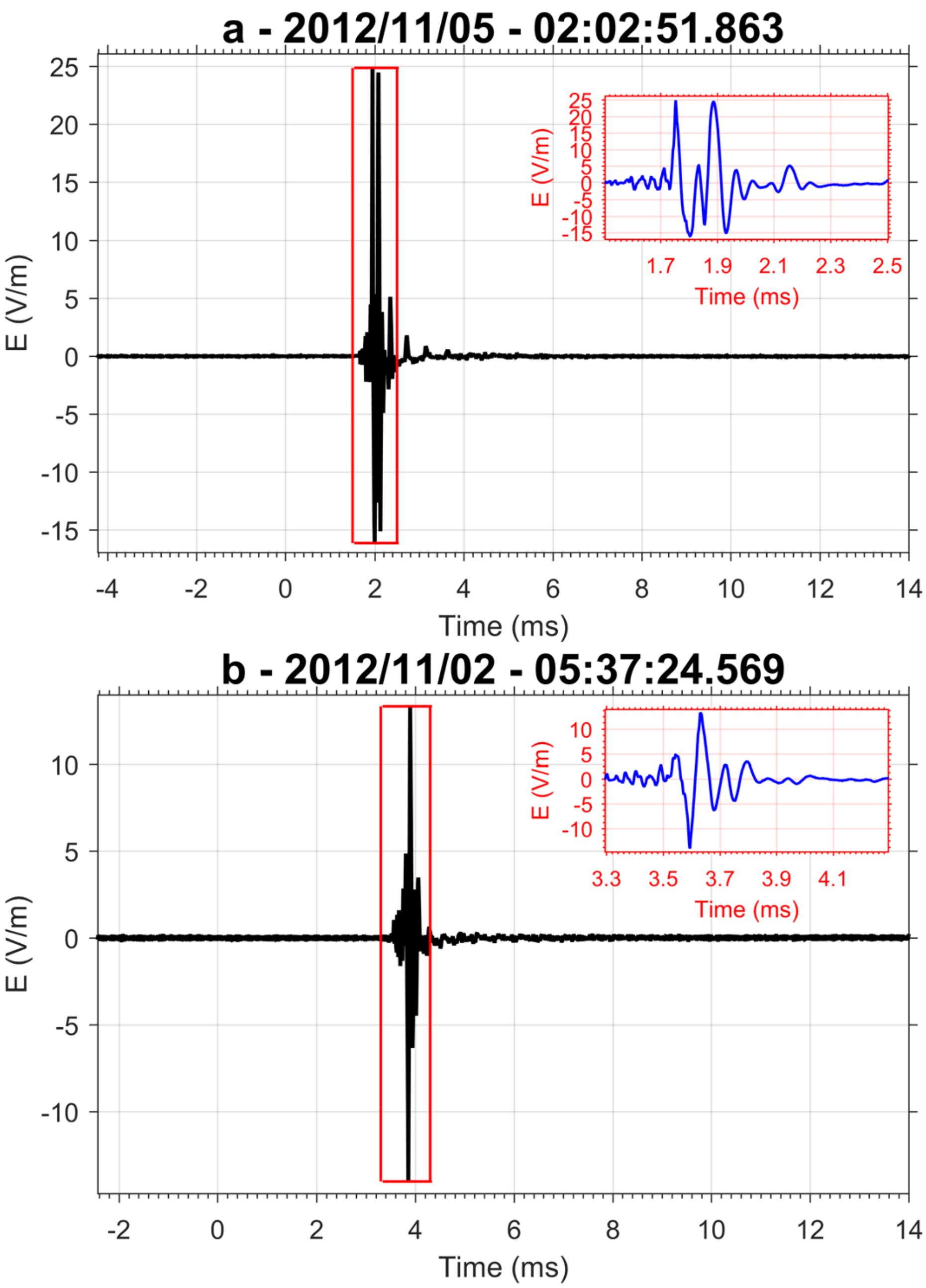

Supplementary Figure 4: **Superbolt ground-based electric field waveforms**. Waveform of (a) the superbolt presented in Figure 2e of the main text and (b) the superbolt presented in Figure 2f of the main text. The waveform in the red box is zoomed in the red inset.

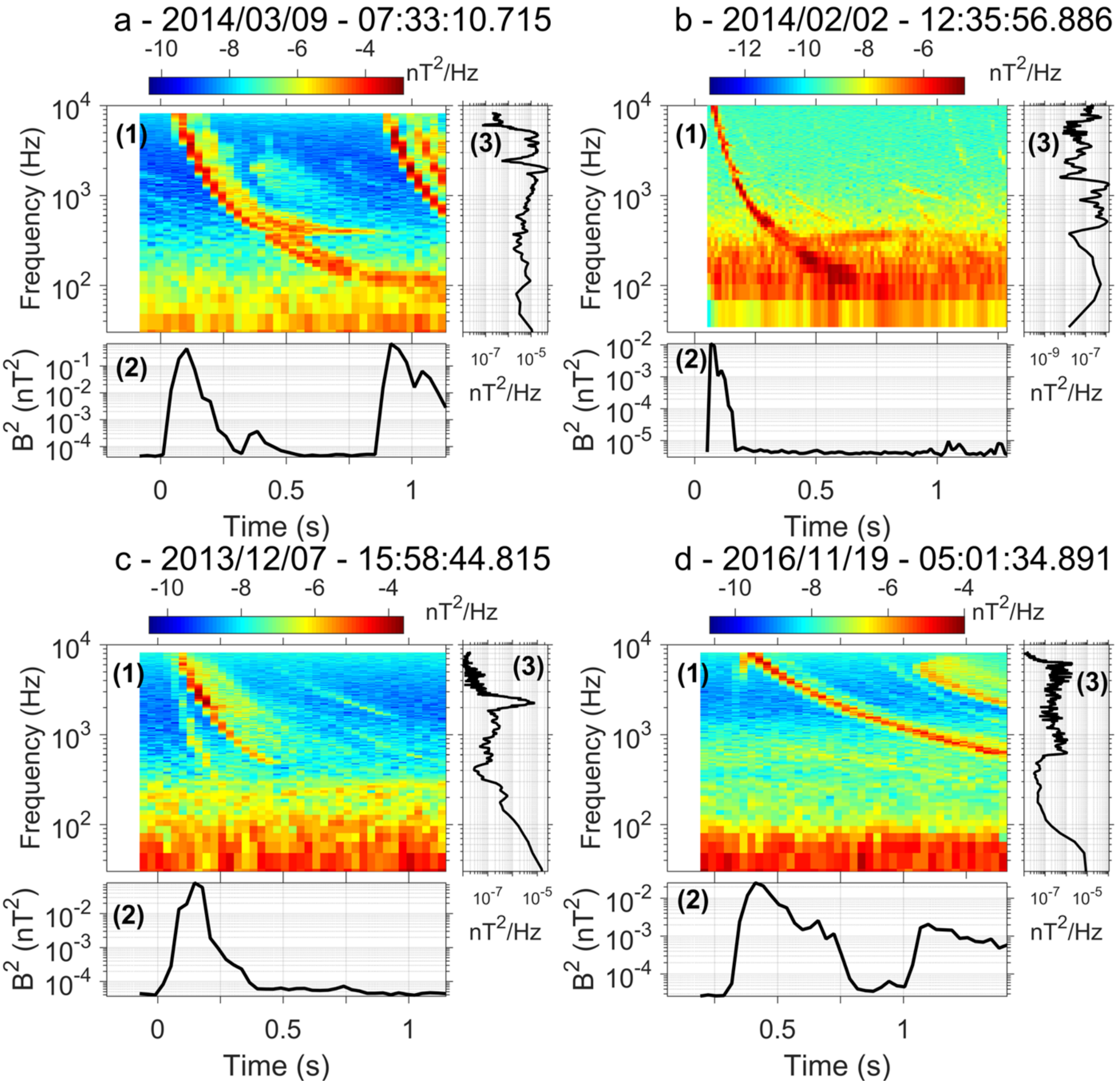

Supplementary Figure 5: **Superbolt magnetic field power spectrum density.** (image 1 of each 4 panels) Magnetic field power spectrum density (PSD in nT^2/Hz) of the 4 superbolts presented in Figure 2 (a-d) of the main text and measured in burst mode in space (with information gathered in Supplementary Table 2). (image 2 of each 4 panels) Evolution of the magnetic field intensity (in nT^2) of these superbolts. (image 3 of each 4 panels) The temporal mean of the PSD (in nT^2/Hz).

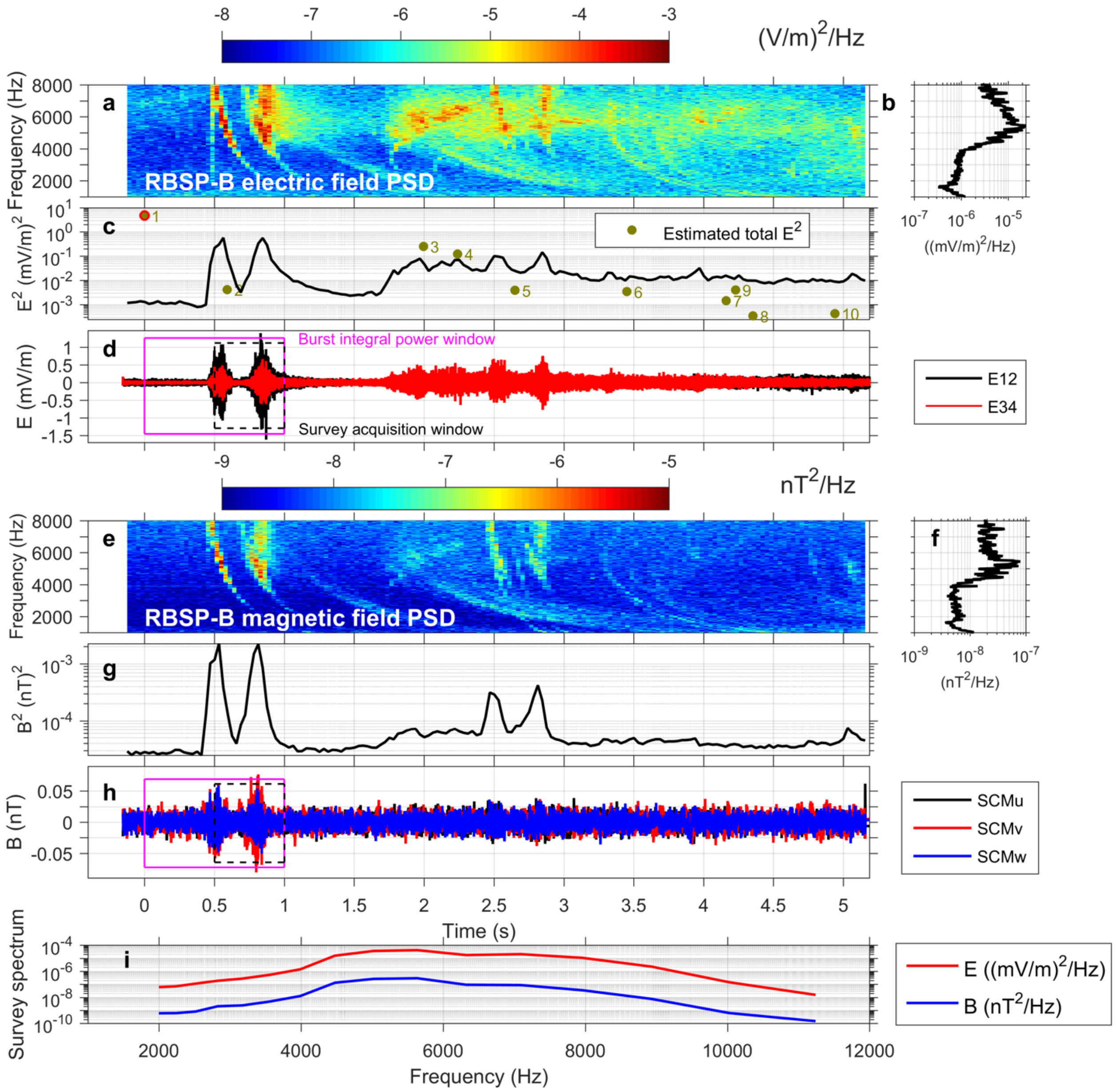

Supplementary Figure 6: **All space measurements of the 2013/01/23 superbolt**. Figure illustrating the analysis method followed for each superbolt, here restricted to space measurements. The superbolt is detected by WWLLN at t=0 on 2013/01/23-17.43.55.121 UTC. We display the Van Allen Probes burst mode measurements of (a) the electric field power spectral density (PSD in in $mV^2/m^2/Hz$) with (b) its time-average spectrum, (c) the squared electric field, (d) the electric field waveform, (e) the magnetic field PSD in pT^2/Hz with (f) its spectrum, (g) the squared magnetic field, (h) the magnetic field waveform, (i) the survey electric and magnetic field time-integrated power spectral density (within the black window). The electromagnetic field PSD has a characteristic descending tone shape (between t~0.4-0.6 s) but shows a second wave at t=0.6s that is the reflection of a secondary wave of the superbolt. The superbolt frequency reaches 400 Hz (deep in the whistler-mode hiss wave band) after 2s (e). The sharp rising tone just prior to the main whistler profile in (a) is an anti-aliasing filter effect with a fold over of the power above the top frequency. This effect is a classic measurement feature also visible in Figure 2a, 2c, 2d, 3a of the main text and in Supplementary Figure 5d.

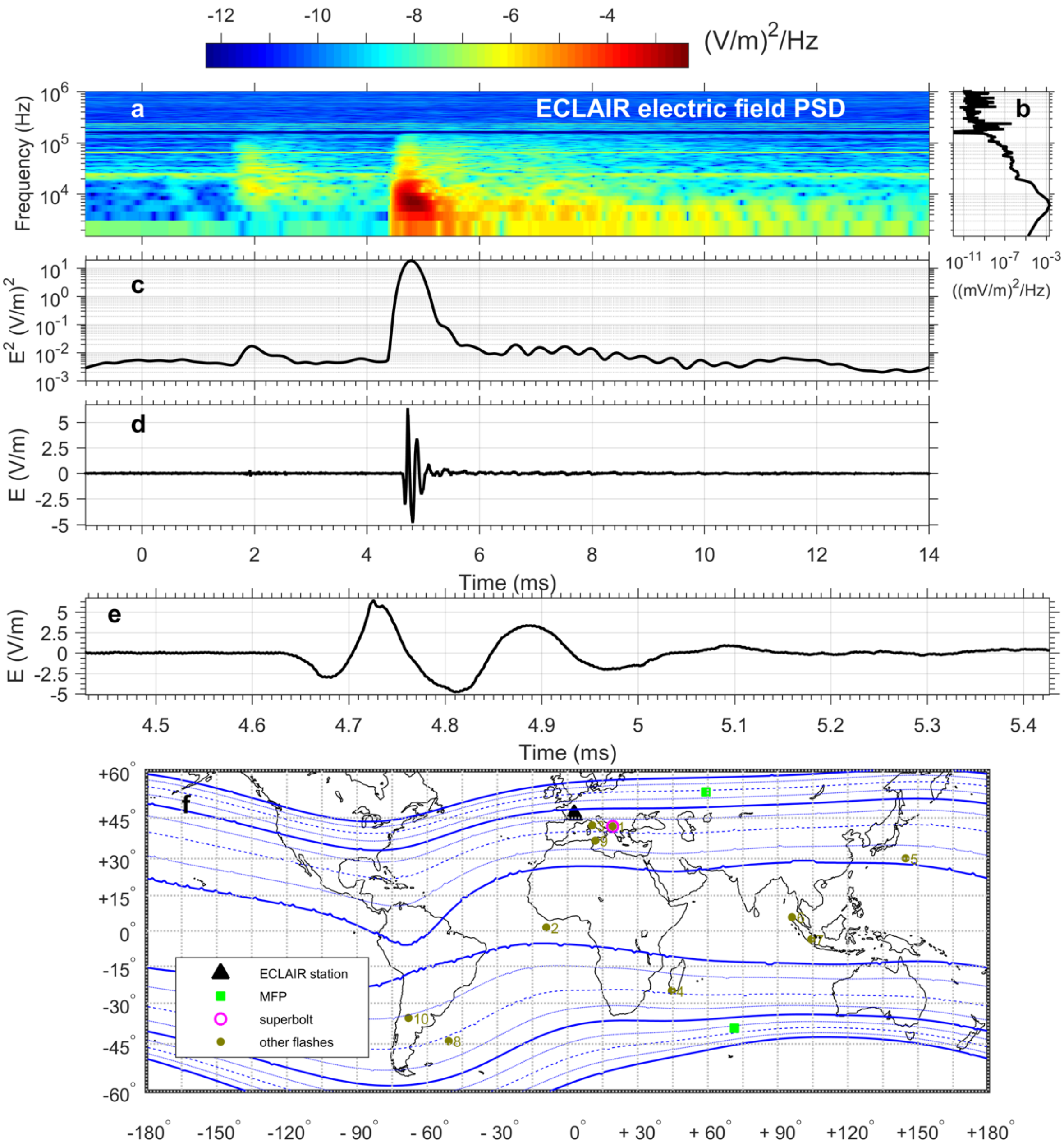

Supplementary Figure 7: **All ground-based information and measurements of the 2013/01/23 superbolt.** Figure illustrating the analysis method followed for each superbolt, here restricted to ground-based measurements. On the ground, we display (a) the electric field PSD in in $V^2/m^2/Hz$ with (b) its time-average spectrum, (c) the squared electric field, (d, e) the electrical field, (f) a map with (pink) the superbolt location, (green) Van Allen Probes magnetic footprints (MFP), and all other WWLLN-detected flashes (#2-10) that occurred within the 5 seconds of the burst measurements (with their intensity also estimated in panel (c) of Supplementary Figure 6 and all listed in Supplementary Table 3). The superbolt PSD at t=0.5s in Supplementary Figure 6(a,e) is not perturbed by another strong lightning (cf. intensity reported in Supplementary Figure 6c). The first peak on the ground is symmetric (a,e). The map itself is made with ©Matlab Mapping Toolbox.

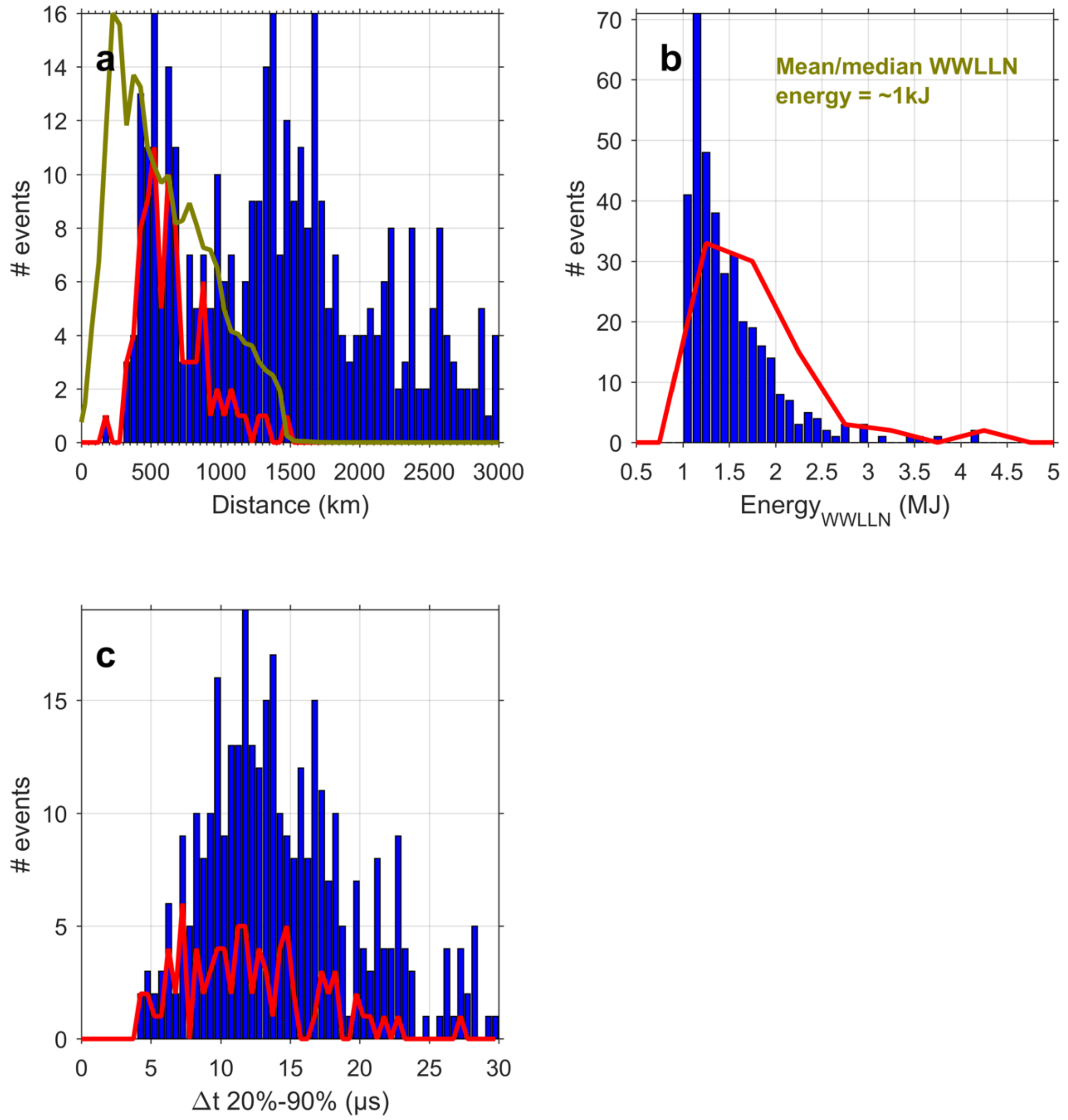

Supplementary Figure 8: **Complementary statistics of superbolt ground measurements.** Data from (blue) ECLAIR and (red) from both ECLAIR and MTRG stations are compared with (green) either the normalized statistics of regular lightning flashes measured by ECLAIR ground stations (from 3349 events and normalized to the maximum of superbolt statistics) or WWLLN: (a) superbolt distance, (b) WWLLN energy (see Figure 2 in [6] for a comparison with the full WWLLN lightning energy distribution), (c) rise time (20-90%). Rise time (20%-90%) are not generated for all the 3349 ECLAIR events since the detection of the 20% peak can be tedious, sometimes impossible, in an automatic way for the less powerful lightning strokes. Reference values of the (10%-90%) rise time for typical lightning are available in [11].

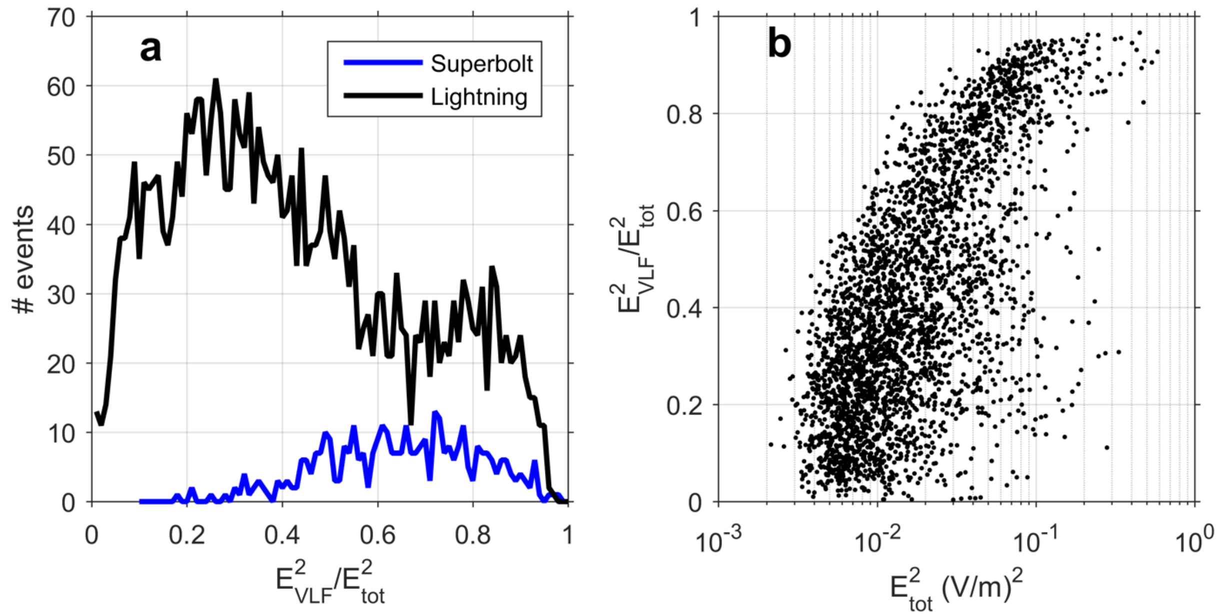

Supplementary Figure 9: **Superbolt VLF and total electromagnetic power on the ground.** (a) Distribution of the ratio of the mean intensity measured on the ground and computed in the VLF range (2-12 kHz) with the total mean on the ground (2 kHz – 5 MHz) for (red) all superbolts and (black) typical lightning strokes (all ECLAIR data). The median of these distributions is 38% for lightning and 68% for superbolt. (b) The latter ratio for typical lightning strokes versus the total intensity showing a monotone increase with the intensity and explaining powerful events, such as superbolts, emit strongly in the VLF range.

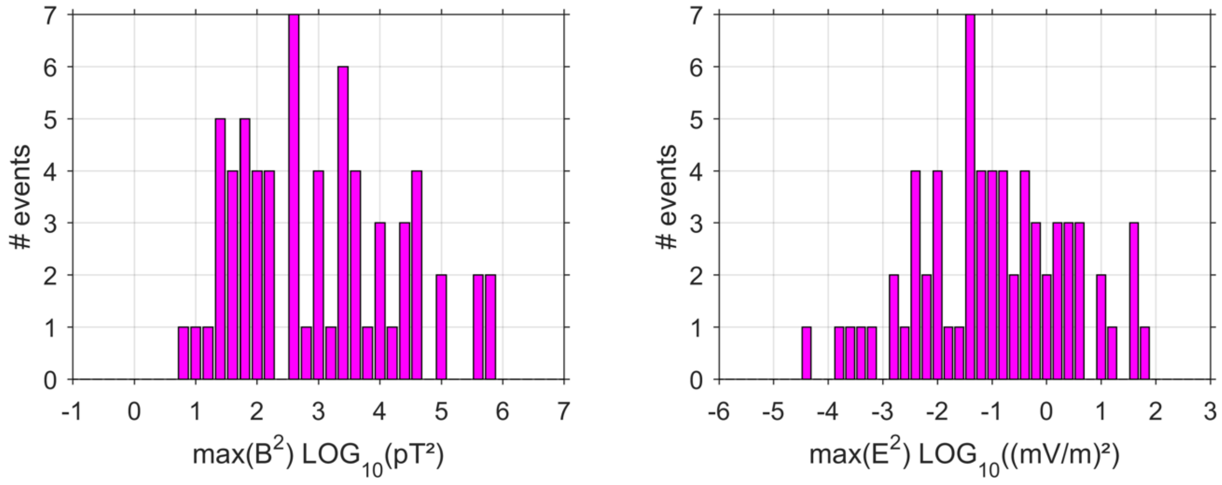

Supplementary Figure 10: **Statistics of superbolt maximum power**. Maximum complementary statistics of Van Allen Probes maximum (left) magnetic and (right) electric field squared from (pink) burst mode measurements. The mean [median] of the magnetic (electric) peak power is 38149 pT^2 (3.6 mV^2/m^2) [696 pT^2 (0.1 mV^2/m^2)], exceeding by a factor 9.8 (13.7) [6.4 (7.2)] the mean [median] of the magnetic (electric) averaged power plotted in Figure 6 (bottom row) of the main text. For calibration purpose, peak power can be rescaled at any distance from the scaling laws given in Figure 4 (b,c) of the main text. The mean [median] distance between WWLLN superbolt

location and the closest magnetic footprint of the Van Allen Probes is 5924 km [5023 km].

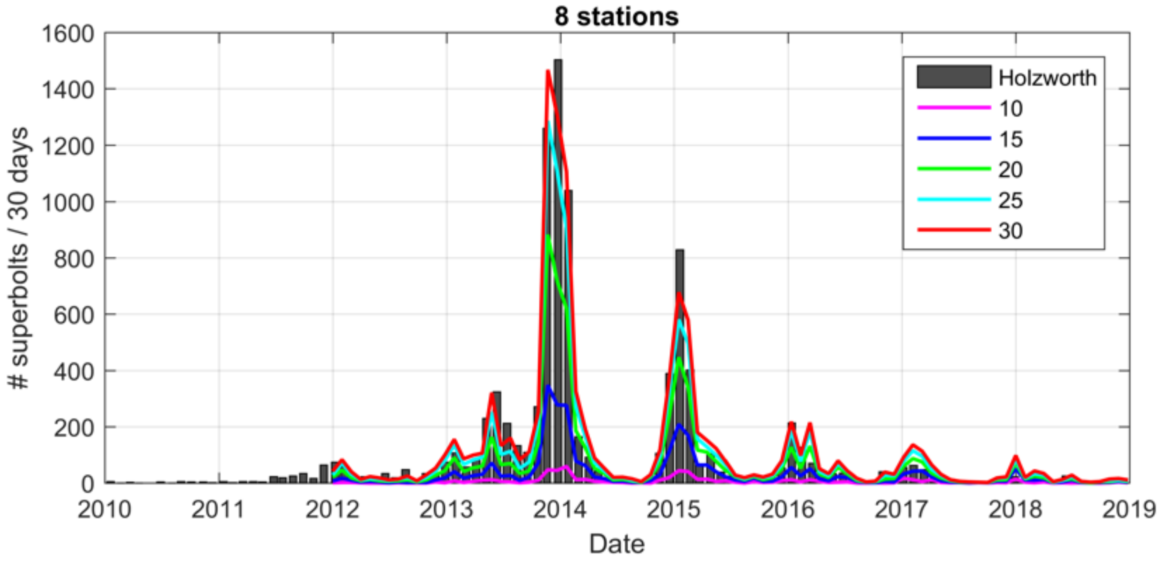

Supplementary Figure 11: **WWLLN localization of superbolts.** Number of localized superbolts when received simultaneously by 8 stations or more within (colored lines) a series of WWLLN residual limits in 2010-2019. Superbolt counts in this study (red) are compared with (grey histograms) statistics in [6] with a minimum station number of seven, and estimated residual of 25 μs. The total numbers of superbolts is (grey histograms) 9290 events and (red line) 10,724 events.

Supplementary References

1. Farges, T., and Blanc, E. Lightning and TLE electric fields and their impact on the ionosphere, *C. R. Phys.*, **12**, 171–179, doi:10.1016/j.crhy.2011.01.013 (2011).

2. Kolmašová, I., Santolík, O., Farges, T., Cummer, S. A., Lán, R., and Uhlíř, L. Subionospheric propagation and peak currents of preliminary breakdown pulses before negative cloud-to-ground lightning discharges, *Geophysical Research Letters*, **43**, 1382-1391, doi:10.1002/ 2015GL067364 (2016).

3. Mauk, B. H., N. J. Fox, S. G. Kanekal, R. L. Kessel, D. G. Sibeck and A. Ukhorskiy. Science Objectives and Rationale for the Radiation Belt Storm Probes Mission, *Space Science Reviews*, **179**(1–4), 3–27, doi: 10.1007/s11214-012-9908-y (2013).

4. Wygant, J. R., J. W. Bonnell, K. Goetz, R. E. Ergun, F. S. Mozer, S. D. Bale, M. Ludlam, P. Turin, P. R. Harvey, R. Hochmann, K. Harps, G. Dalton, J. McCauley, W. Rachelson, D. Gordon, B. Donakowski, C. Shultz, C. Smith, M. Diaz-Aguado, J. Fischer, S. Heavner, P. Berg, D. M. Malsapina, M. K. Bolton, M. Hudson, R. J. Strangeway, D. N. Baker, X. Li, J. Albert, J. C. Foster, C. C. Chaston, I. Mann, E. Donovan, C. M. Cully, C. A. Cattell, V. Krasnoselskikh, K. Kersten, A. Brenneman, and J. B. Tao. The Electric Field and Waves Instruments on the Radiation Belt Storm Probes Mission. *Space Science Reviews*, **179**,183–220. Doi: 10.1007/s11214-013-0013-7 (2013).

5. Kletzing, C. A., W. S. Kurth, M. Acuna, R. J. MacDowall, R. B. Torbert, T. Averkamp, D. Bodet, S. R. Bounds, M. Chutter, J. Connerney, D. Crawford, J. S. Dolan, R. Dvorsky, G. B. Hospodarsky, J. Howard, V. Jordanova, R. A. Johnson, D. L. Kirchner, B. Mokrzycki, G. Needell, J. Odom, D. Mark, R. Pfaff, J. R. Phillips, C. W. Piker, S. L. Remington, D. Rowland, O. Santolik, R. Schnurr, D. Sheppard, C. W. Smith, R. M. Thorne, and J. Tyler. The Electric and Magnetic Field Instrument Suite and Integrated Science (EMFISIS) on Van Allen Probes. *Space Science Reviews*, **179**, 127–181. Doi: 10.1007/s11214-013-9993-6 (2013).

6. Holzworth, R. H., McCarthy, M. P., Brundell, J. B., Jacobson, A. R., & Rodger, C. J. Global distribution of superbolts. *Journal of Geophysical Research: Atmospheres*, **124**, 9996–10,005. https://doi.org/10.1029/ 2019JD030975 (2019).

7. Hutchins, M. L., R. H. Holzworth, C. J. Rodger, and J. B. Brundell, Far-field power of lightning strokes as measured by the World Wide Lightning Location Network, *J. Atmos. Oceanic Technol.*, **29**, 1102–1110, doi:10.1175/JTECH-D-11-00174.1 (2012).

8. Burkholder, B. S., M. L. Hutchins, M. P. McCarthy, R. F. Pfaff, and R. H. Holzworth. Attenuation of lightning-produced sferics in the Earth-ionosphere waveguide and low-latitude ionosphere, *J. Geophys. Res. Space Physics*, **118**, 3692–3699, doi:10.1002/jgra.50351 (2013).

9. Burgesser, R. E. Assessment of the World Wide Lightning Location Network (WWLLN) detection efficiency by comparison to the Lightning Imaging Sensor (LIS). *Quarterly Journal of the Royal Meteorological Society*, **143**(708), 2809–2817. https://doi. org/10.1002/qj.3129 (2017)

10. Rudlosky, S. D., & Shea, D. T. Evaluating WWLLN Performance Relative to TRMM/LIS *Geophysical Research Letters*, **40**, 2344–2348 https://doi.org/10.1002/grl.50428 (2013)

11. Rakov, V. A., and M. A. Uman, Lightning: Physics and Effects (Cambridge Univ. Press, Cambridge, 2007).
